# Preparation and Evaluation of Radiolabeled Porphyrin-Functionalized Lipid Nanodroplets for Cancer Theranostics

**DOI:** 10.3390/molecules31071114

**Published:** 2026-03-27

**Authors:** Nur Izni Binti Ramzi, Kisa Tamamura, Masayuki Munekane, Kenji Mishiro, Takeshi Fuchigami, Xiaojun Hu, Renata Jastrząb, Seigo Kinuya, Kazuaki Ninomiya, Kazuma Ogawa

**Affiliations:** 1 Graduate School of Medical Sciences, Kanazawa University, Kakuma-machi, Kanazawa 920-1192, Ishikawa, Japan; 2 Center for Molecular Recognition and Biosensing, School of Life Sciences, Shanghai University, Shanghai 200444, China; 3 Faculty of Chemistry, Adama Mickiewicz University in Poznan, Uniwersytetu Poznańskiego 8, 61-614 Poznan, Poland; 4 Department of Nuclear Medicine, Kanazawa University Hospital, Kanazawa University, Takara-machi 13-1, Kanazawa 920-8641, Ishikawa, Japan; 5 Faculty of Biological Science and Technology, Institute of Science and Engineering, Kanazawa University, Kakuma-machi, Kanazawa 920-1192, Ishikawa, Japan

**Keywords:** cancer theranostics, nanodroplets, porphyrin, sonodynamic therapy, single photon emission computed tomography

## Abstract

[^111^In]In-diethylenetriaminepentaacetic acid-5,10,15,20-tetraphenylporphyrin ([^111^In]In-DTPA-TPP) nanodroplets were developed for cancer theranostics, featuring ultrasound-sensitive properties. The designed nanodroplets that encapsulate the low-boiling-point liquid perfluorocarbon and IR-780 iodide, a near-infrared fluorescent dye, with surface conjugation of ^111^In-labeled porphyrin derivative, were synthesized and evaluated by in vitro and in vivo experiments. The cellular uptake of [^111^In]In-DTPA-TPP nanodroplets was significantly higher than that of control nanodroplets without TPP. Biodistribution experiments revealed greater tumor accumulation in mice injected with [^111^In]In-DTPA-TPP nanodroplets than in those injected with control nanodroplets lacking TPP. Additionally, the accumulation of [^111^In]In-DTPA-TPP nanodroplets in the tumor was visualized by single-photon emission computed tomography. Sonodynamic therapeutic experiments revealed that DTPA-TPP nanodroplets at 10 µmol total lipids/kg weight with a single ultrasound irradiation onto the tumor area significantly inhibited tumor growth. These results indicate that [^111^In]In-DTPA-TPP nanodroplets would be promising cancer theranostic agents.

## 1. Introduction

The adoption of nanosized drug delivery systems has rapidly evolved as a treatment modality for various diseases [1,2]. Nanodrug formulations have demonstrated significant advantages over their free drug counterparts, including improved pharmacokinetics, enhanced efficacy, reduced toxicity, and increased target lesion selectivity [3,4]. The enhanced permeability and retention (EPR) effect governs the selective passive accumulation of nanoparticles in tumors [5]. Irregularly formed blood vessels during tumorigenesis, leading to “leaky” tumor vasculatures, cause the EPR effect [6]. This malformation enhances the permeability of large particles such as nanoparticles, macromolecules, liposomes, and other soluble particles into the tumor. Simultaneously, extravasated nanoparticles are retained in tumor tissues due to their slow expulsion caused by the poorly formed lymphatic systems [7]. The extent of tumor accumulation is strongly influenced by the physicochemical properties of nanocarriers, including particle size and shape. Nanoparticles in the range of approximately 20–200 nm are generally considered favorable for prolonged circulation and enhanced tumor retention [8], whereas larger particles show limited extravasation and smaller particles may undergo rapid renal clearance. Additionally, non-spherical particles have been reported to exhibit distinct vascular dynamics compared with spherical nanocarriers [9]. Passive targeting via the EPR effect using liposomal drugs has only been partially successful in clinical trials despite favorable preclinical results [10,11]. Therefore, the delivery of nanodrugs to cancers based on the EPR effect alone may be insufficient [12,13]. Additional strategies are required to improve the targeted drug delivery of nanomedicines. Porphyrins are highly reactive photosensitizers equipped with robust therapeutic and diagnostic properties. Reportedly, porphyrin uptake relies on its interaction with various proteins, such as transferrin [14], hemopexin [15], low-density lipoproteins [16], and a porphyrin transporter via heme carrier protein 1 [17]. Various studies suggested the preferential uptake of porphyrins into cancer cells in comparison to normal cells, although elucidation of their exact mechanism of uptake remains underway [18]. Therefore, surface conjugation of porphyrins to nanomaterials could improve the selective overall nanoparticle localization toward cancers.

Photodynamic therapy (PDT) is a useful treatment that uses light to target tumor tissues while minimizing damage to normal tissues [19]. However, the efficacy of PDT is significantly reduced by hypoxia in solid tumors because PDT produces reactive oxygen species (ROS), requiring remarkable oxygen consumption. Perfluorocarbon (PFC) has been recently used as an oxygen carrier to overcome this problem [20]. Phase shift nanodroplets that contain a low-boiling-point liquid PFC core have been used for ultrasound imaging agents and drug delivery carriers [21]. Nanodroplets, as oxygen carriers, are also applicable for PDT by combining them with photosensitizers. Nanodroplets made from lipid shells containing a perfluorohexane (PFH) core with a photosensitizer IR-780 dye (2-[2-[2-chloro-3-[(1,3-dihydro-3,3-dimethyl-1-propyl-2*H*-indol-2-ylidene)ethylidene]-1-cyclohexen-1-yl]ethenyl]-3,3-dimethyl-1-propylindolium iodide) demonstrated potent therapeutic effects with near-infrared light irradiations [22]. Although PDT has been reported in numerous clinical treatments of cancers [23], a notable drawback is the low permeability of light for photosensitizer photoactivation in deep tissues, which limits PDT application to superficial lesions [24]. Recently, ultrasound-triggered agents for sonodynamic therapy (SDT) have gained attention due to their non-invasiveness and ability to penetrate deeply seated tumors [25]. Furthermore, SDT using nanoparticles that contain PFC and sonosensitizers is a preferable strategy against deep tumor nidus [26]. In these studies, in vivo experiments by SDT demonstrated efficient tumor inhibition.

**Figure 1:**
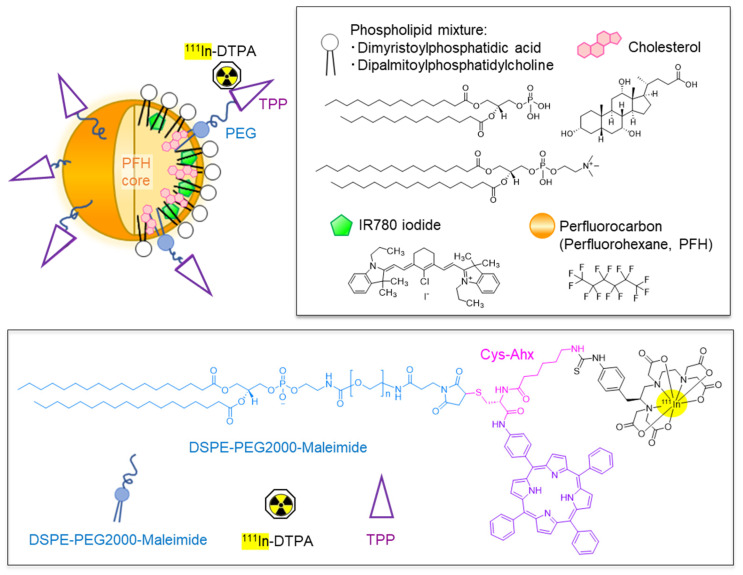
Structure and design of a nanodroplet-based theranostic agent. PFH and IR-780 dye are co-encapsulated by phospholipids and cholesterol. ^111^In-labeled porphyrin derivative is conjugated on the surface of nanodroplets.

In this context, the development of multifunctional nanoplatforms that integrate radionuclide imaging capability with ultrasound-triggered therapy represents a promising strategy for precise cancer theranostics. Namely, the present study prepared ultrasound-sensitive nanodroplets, which encapsulate PFH and IR-780 dye, with surface conjugation of Indium-111 (^111^In)-labeled porphyrin derivative for cancer theranostics (Figure 1). Here, diethylenetriaminepentaacetic acid (DTPA) was used as a chelator because DTPA can form a stable complex with ^111^In (t_1/2_ = 2.83 d) capable of single photon emission computed tomography (SPECT) imaging. Additionally, polyethylene glycol (PEG) was integrated into the nanodroplet to prolong its blood circulation time [27]. This nanodroplet design uses sonoluminescence, which can consequently convert the molecular oxygen dissolved in PFH to form the highly reactive singlet oxygen species [28,29], to activate sonosensitizer IR-780 dye [30] upon ultrasound irradiation. The synergistic effect of the PFC and sonosensitizer is a lethal combination for sonodynamic therapy. Additionally, porphyrin functionalization to the nanodroplets could potentially improve the biodistribution and uptake of the overall nanodroplets toward tumors.

## 2. Results

### 2.1. Synthesis of Intermediates and Precursor

**Scheme 1:**
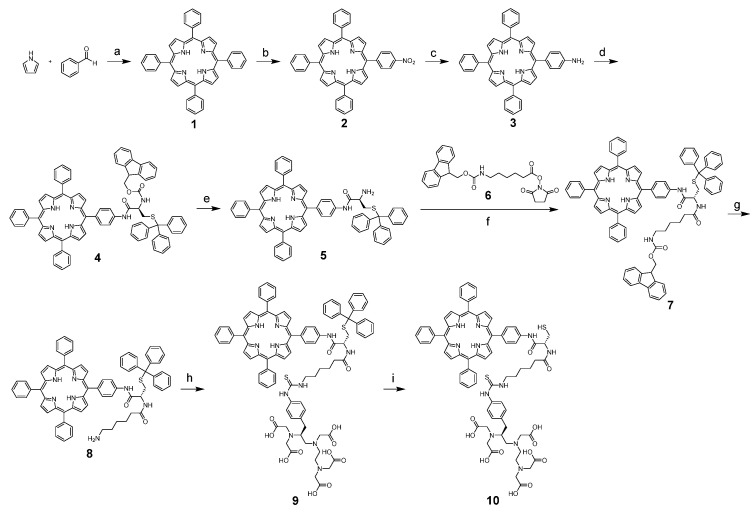
Synthesis of precursor DTPA-TPP (**10**). Reagents and conditions: (a) propionic acid, reflux, 24 h; (b) TFA, NaNO_3_, rt, 3 min; (c) 12 M HCl, tin (II) chloride, 65 °C, 1 h; (d) DMF, *N*-Fmoc-*S*-trityl-l-cysteine, HOAT, HATU, DIPEA, rt, 1 h; (e) DMF, piperidine, rt, 1 h; (f) DMF, 40 °C, 3 h; (g) DMF, piperidine, rt, 1 h; (h) DMF, *p*-SCN-Bn-DTPA, DIPEA, rt, 1 h; (i) TFA, water, TIS, rt, 1 h.

We first synthesized the precursor DTPA-TPP (**10**) (Scheme 1) for nanodroplet synthesis and radiolabeling. Acid-catalyzed pyrrole and aldehyde condensation reaction under an aerobic environment, known as the Adler-Longo method, generated TPP (**1**) in 2.9% yield. Compound **1** was nitrated to generate TPP-NO_2_ (**2**) in a 23% yield, followed by its reduction to produce TPP-NH_2_ (**3**) in an 89% yield. Fmoc-*S*-trityl-l-cysteine-TPP (**4**) was obtained by hexafluorophosphate azabenzotriazole tetramethyl uranium (HATU) coupling of **3** and *N*-Fmoc-*S*-trityl-l-cysteine using *N*,*N*-diisopropylethylamine (DIPEA) as a base in a 94% yield. Fmoc deprotection of **4** under basic conditions yielded *S*-trityl-l-cysteine-TPP (**5**) in 91%. The carboxy group in Fmoc-6-aminohexanoic acid was then converted to an *N*-hydroxysuccinimide (NHS) ester by 1-ethyl-3-(3-dimethylaminopropyl)carbodiimide (EDCI) coupling to obtain Fmoc-6-aminohexanoic acid NHS ester (**6**) in an 81% yield. Fmoc-6-Ahx-*S*-trityl-l-cysteine-TPP (**7**) was obtained by conjugating **5** and **6** in a 44% yield. Fmoc deprotection of **7** in 20% piperidine generated 6-Ahx-*S*-trityl-l-cysteine-TPP (**8**) in a 58% yield. DTPA-6-Ahx-*S*-Trityl-l-cysteine-TPP (**9**) was obtained by conjugating *p*-SCN-Bn-DTPA and **8** using DIPEA as a base in 66% yield. Finally, trityl deprotection under acidic conditions generated DTPA-TPP (**10**) in a 95% yield.

**Scheme 2:**
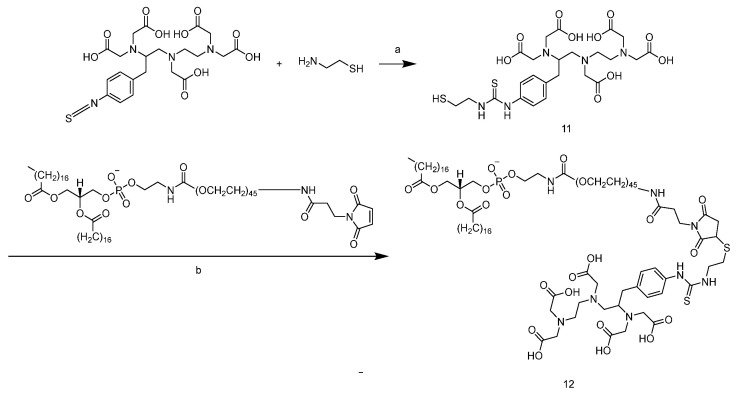
Synthesis of precursor DTPA-cysteamine-PEG_2000_ (**12**). Reagents and conditions: (a) DMF, TEA, rt, 1 h; (b) 0.1 M PB, 0.15 M NaCl, 2 mM EDTA, TEA, rt, 24 h.

Moreover, we synthesized the precursor DTPA-cysteamine-PEG_2000_ (**12**) (Scheme 2) for preparing control nanodroplets to evaluate the influence of porphyrin-conjugated nanodroplets. DTPA-cysteamine (**11**) was obtained by conjugating *p*-SCN-Bn-DTPA and cysteamine in 66% yield. Finally, **11** was conjugated with DSPE-PEG_2000_-maleimide using TEA as a base to afford **12** in a 75% yield.

### 2.2. Nanodroplet Synthesis

DTPA-cysteamine-nanodroplets and DTPA-TPP-nanodroplets were synthesized by using the thin film hydration technique. The dynamic light scattering instrument with a 540 nm laser beam, performed at 25 °C, was used to assess the particle sizes of both nanodroplets. The particle sizes of DTPA-cysteamine-nanodroplets and DTPA-TPP-nanodroplets were 205.7 ± 36.3 and 208.0 ± 36.6 nm, respectively (Figure S2).

### 2.3. Nanodroplet Radiolabeling

[^111^In]In-DTPA-cysteamine-nanodroplets and [^111^In]In-DTPA-TPP-nanodroplets were synthesized in 91.4% and 77.7% radiochemical yields, respectively. The radiochemical purities of both products were >97%.

### 2.4. Cellular Uptake Experiments

**Figure 2:**
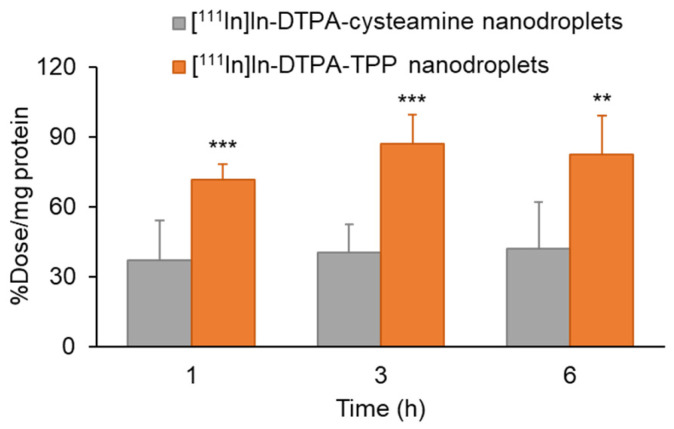
Cellular uptake study. Time-dependent accumulation of [^111^In]In-DTPA-cysteamine and [^111^In]In-DTPA-TPP nanodroplets in Colon 26 cells. Data were presented as mean ± SD for six samples. ** *p* < 0.01, *** *p* < 0.001 vs. [^111^In]In-DTPA-cysteamine nanodroplets.

Figure 2 shows the cellular uptake of [^111^In]In-DTPA-cysteamine and [^111^In]In-DTPA-TPP nanodroplets in Colon 26 cells. The uptake of control nanodroplets ([^111^In]In-DTPA-cysteamine nanodroplets) was relatively similar throughout all time points. Meanwhile, [^111^In]In-DTPA-TPP nanodroplets steadily increased up to 3 h and then eventually plateaued at 6 h. Overall, the uptake of [^111^In]In-DTPA-TPP nanodroplets was significantly higher than that of the control nanodroplets at all time points.

### 2.5. Biodistribution Experiments and SPECT/CT Imaging

Table 1 summarizes the biodistributions of [^111^In]In-DTPA-cysteamine and [^111^In]In-DTPA-TPP nanodroplets in Colon 26 tumor-bearing mice. Table S1 summarizes the biodistributions of [^111^In]InCl_3_ in ddY mice. High radioactivity in the liver and spleen was observed, indicating a reticuloendothelial clearance for both nanodroplets [31]. Additionally, the tumor uptakes of [^111^In]In-DTPA-TPP nanodroplets at 1, 6, and 24 h were 1.62, 3.39, and 2.76% ID/g, respectively, which were significantly higher than those of [^111^In]In-DTPA-cysteamine nanodroplets.

**Figure 3:**
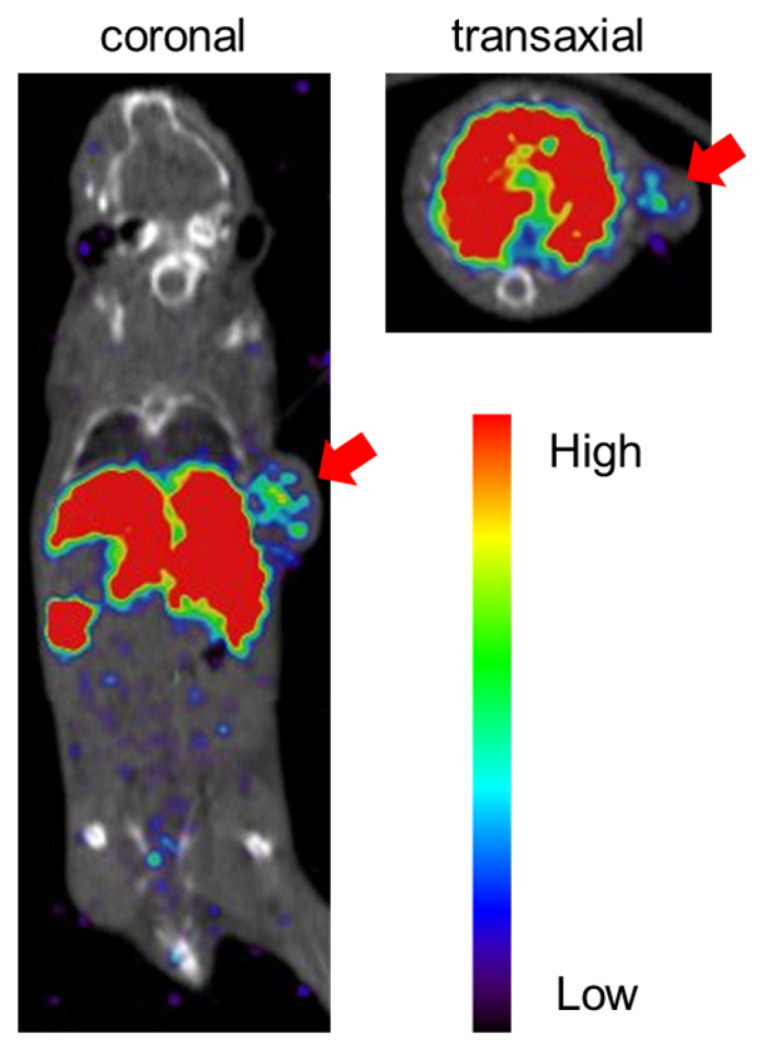
SPECT/CT imaging. Colonal and transaxial SPECT/CT images of Colon 26 tumor-bearing mouse at 24 h after administration of [^111^In]In-DTPA-TPP nanodroplets. Red arrow indicates tumor.

Figure 3 shows the SPECT/CT images in Colon 26 tumor-bearing mice after [^111^In]In-DTPA-TPP nanodroplets injection. The time point for SPECT imaging was set at 24 h postinjection of [^111^In]In-DTPA-TPP-nanodroplets, based on the biodistribution results. The tumor was visualized, although a prominently high accumulation of radioactivity was observed in the liver and spleen.

### 2.6. In Vivo SDT Assessment

**Figure 4:**
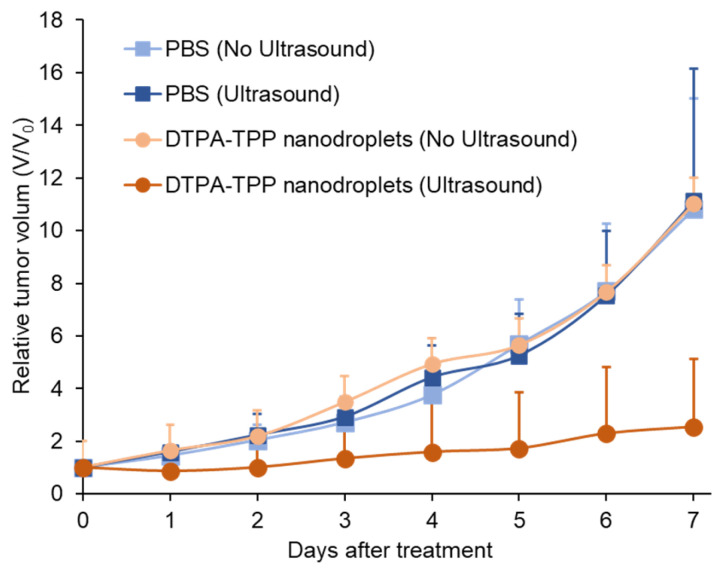
Relative tumor volumes (V/V_0_) of Colon 26 tumor-bearing mice injected with PBS as the control group, accompanied by ultrasound and DTPA-TPP nanodroplets, administered at a dose of 10 μmol of total lipids/kg, accompanied by ultrasound. Ultrasound irradiation (1 MHz, 2.4 W/cm^2^, 50% duty cycle) was performed once at 6 h postinjection. Each value represents mean ± SD for four mice.

**Figure 5:**
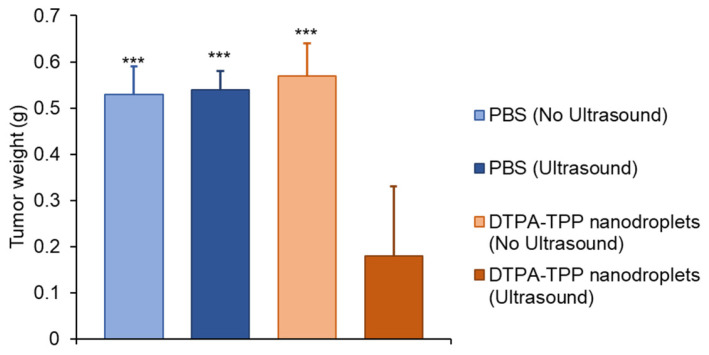
Tumor weight (g) on day 7 postinjection for PBS- or DTPA-TPP nanodroplets-treated mice, with or without ultrasound irradiation. Each value represents mean (SD) for four mice. *** *p* < 0.001 vs. DTPA-TPP nanodroplets with ultrasound irradiation.

In vivo SDT experiments were performed to evaluate the therapeutic efficacy of DTPA-TPP nanodroplets at 10 μmol of total lipids/kg by light, ultrasound, and a combination of both at 6 h postinjection in Colon 26 tumor-bearing mice. Figure 4 and Table S2 summarize the tumor sizes expressed as relative volume (V/V_0_) for 7 days postinjection. The weight of each tumor on both shoulders of mice in all groups was measured on day 7 postinjection and expressed as tumor weight (g) in Figure 5. Additionally, the mice’s body weight was also examined throughout the study duration (Figure S3). Tumor growth in mice treated with DTPA-TPP nanodroplets at 6 h postinjection was inhibited after ultrasound irradiation (Figure 4). Meanwhile, tumors in phosphate-buffered saline (PBS) with or without ultrasound- and DTPA-TPP nanodroplets without ultrasound-treated groups continuously grew throughout the study duration. These results corresponded with the tumor weights on day 7 postinjection, wherein tumors treated with DTPA-TPP nanodroplets and ultrasound irradiation weighed significantly lower than those without ultrasound irradiation and those of PBS-treated groups. No obvious weight loss was observed throughout the study duration for all groups (Figure S3).

## 3. Discussion

We prepared [^111^In]In-DTPA-TPP-nanodroplets to develop radiolabeled nanodroplets for cancer theranostics. Based on our previous results, radiolabeled TPP derivatives demonstrated promising theranostics applications due to their high tumor accumulation [32]. Therefore, this study designed nanodroplets decorated with TPP as targeting moieties, aimed at improving cancer selectivity, followed by encapsulation of a PFC derivative, PFH, and the sonosensitizer IR-780 dye for SDT.

Although effective tumor targeting has been reported across a broad size range, many nanoparticle-based drug delivery systems are designed within the 20–200 nm range to facilitate EPR-mediated tumor accumulation [8]. In addition, larger nanodroplets can encapsulate a greater amount of perfluorocarbon per particle, which may enhance acoustic droplet vaporization and cavitation effects. Considering these factors, our study prepared nanodroplets with an approximate particle size of 200 nm. Subsequently, the lipid composition was carefully selected to achieve a balance between structural stability and ultrasound responsiveness of the nanodroplets. A relatively high cholesterol fraction was incorporated to strengthen membrane packing and improve nanodroplet stability. Furthermore, 1,2-dimyristoyl-*sn*-glycero-3-phosphatidic acid (DMPA) and 1,2-dipalmitoyl-*sn*-glycero-3-phosphocholine (DPPC), which possess relatively high phase transition temperatures, help maintain membrane rigidity under physiological conditions while still allowing ultrasound-induced local heating to trigger phase transition and controlled release. This design provides both mechanical robustness and acoustic responsiveness, which are required for effective sonodynamic therapy.

In vitro cellular uptake experiments for [^111^In]In-DTPA-TPP and [^111^In]In-DTPA-cysteamine nanodroplets were performed. The cellular uptake of [^111^In]In-DTPA-TPP nanodroplets at all time points was significantly higher than that of the control nanodroplets, [^111^In]In-DTPA-cysteamine nanodroplets, indicating that surface conjugation of porphyrins influenced the cellular interaction of the nanodroplets (Figure 2). Additional experiments performed in FBS-containing medium showed reduced cellular uptake compared with serum-free conditions. Furthermore, competitive inhibition experiments using free porphyrin did not significantly suppress nanodroplet uptake. These results suggest that the enhanced cellular uptake is unlikely to be dominated by a specific receptor-mediated mechanism and may instead be related to altered physicochemical interactions induced by porphyrin modification. The biodistribution results of [^111^In]In-DTPA-TPP and [^111^In]In-DTPA-cysteamine nanodroplets (Table 1) demonstrated that the primary clearance pathway of both nanodroplets was by the reticuloendothelial system, as indicated by the high liver and spleen uptake [31]. Similar biodistribution patterns have been reported for clinically approved nanomedicines [33]. Meanwhile, the higher liver uptake and lower kidney uptake of [^111^In]In-DTPA-TPP nanodroplets than those of [^111^In]In-DTPA-cysteamine nanodroplets may reflect the known hepatobiliary behavior of lipophilic porphyrins [18]. Although serum stability assays were not performed in the present study, the relatively low bone uptake observed throughout the study period suggests that significant in vivo release of ^111^In from the DTPA chelator is unlikely, as free ^111^In typically shows progressive bone accumulation (Table S1) [34]. The tumor-to-blood ratios of both radiolabeled nanodroplets increased in a time-dependent manner, whereas tumor-to-muscle ratios peaked at 48 h postinjection. While hepatic and splenic accumulation remains a limitation of nanoparticle-based systems, tumor-selective activation by ultrasound may mitigate off-target therapeutic effects. Additionally, [^111^In]In-DTPA-cysteamine nanodroplets also exhibited tumor accumulation, which is likely attributable to passive targeting via the EPR effect. Notably, a significantly higher tumor accumulation until 24 h postinjection was observed for [^111^In]In-DTPA-TPP nanodroplets compared with control nanodroplets. This difference is consistent with the higher cellular uptake observed in vitro and suggests that porphyrin modification may further enhance tumor accumulation, although the exact mechanism remains to be elucidated. Moreover, the tumor accumulation of [^111^In]In-DTPA-TPP nanodroplets was visualized by SPECT imaging (Figure 3).

SDT experiments were performed in Colon 26 tumor-bearing mice to evaluate the therapeutic efficacy of DTPA-TPP nanodroplets. In vivo treatment studies for DTPA-TPP nanodroplets were performed at 10 μmol of total lipids/kg accompanied by ultrasound irradiation at 6 h postinjection, because the highest accumulation of [^111^In]In-DTPA-TPP nanodroplets was observed in the tumor at 6 h postinjection. Colon 26 cancer cells were inoculated on both shoulders of mice, and ultrasound was irradiated to the left tumor, whereas the right tumor served as a control without ultrasound. The growth of tumors in mice treated with DTPA-TPP nanodroplets accompanied by ultrasound was significantly suppressed compared with that in the PBS-treated groups (Figure 4). Meanwhile, the opposite side of the tumor without ultrasound irradiation grew continuously without therapeutic effects. Additionally, the tumor weights measured at the final observation period in Figure 5 showed that tumors treated with DTPA-TPP nanodroplets, accompanied by ultrasound, weighed significantly less than the other groups, which further corroborated the therapeutic efficacy of the nanodroplets. These results indicated that selective tumor growth inhibition was observed following ultrasound treatment in the presence of DTPA-TPP nanodroplets, suggesting an SDT-mediated therapeutic effect, whereas nanodroplets without ultrasound irradiation did not significantly affect tumor growth. Although mechanistic analyses such as ROS generation and apoptosis pathways were not fully characterized in this study and warrant further investigation, ultrasound administration in the presence of the nanodroplets triggers a series of sonochemical reactions through sonosensitizer (IR-780 dye) excitation [30] followed by PFC emulsion droplet vaporization. This synergistic phenomenon is thought to enable the generation of highly reactive singlet oxygen species and other ROS from the nanodroplets, which may ultimately contribute to cell death. These findings demonstrate the potential sonotoxicity of the nanodroplets and support their feasibility as a proof-of-concept platform for ultrasound-triggered antitumor therapy. In the present study, SDT experiments were conducted using a single lipid dose to demonstrate proof-of-concept therapeutic efficacy. A systematic dose–response evaluation was not performed and should be addressed in future studies to determine the optimal therapeutic dose. Further mechanistic studies will be necessary to elucidate the detailed ROS generation pathways and apoptosis mechanisms associated with SDT induced by the nanodroplets.

Theranostics is an exciting concept that enables personalized medicine. Advanced evaluation of therapeutic drug accumulation in target tissues, such as cancer, using diagnostic imaging may help predict drug effects and inform individualized treatment strategies. Nanoparticles are an attractive platform for theranostics because they can encapsulate various compounds for diagnosis and therapeutic purposes. This study visualized tumor accumulation of [^111^In]In-DTPA-TPP nanodroplets using SPECT imaging, and potent inhibition of tumor growth was observed in SDT experiments. Therefore, [^111^In]In-DTPA-TPP nanodroplets could be a potential theranostic probe for SPECT imaging with SDT.

## 4. Materials and Methods

### 4.1. General

Commercial reagents and solvents were purchased from Sigma-Aldrich (St. Louis, MO, USA), Fujifilm Wako Pure Chemical (Osaka, Japan), Nacalai Tesque, Inc., (Kyoto, Japan), Tokyo Chemical Industry, Co., Ltd., (Tokyo, Japan), Kanto Chemical, Co., Inc. (Tokyo, Japan), Yuka Sangyo Co., Ltd. (Tokyo, Japan), and Macrocyclics Inc, (Dallas, TX, USA) and used without further purification. TLC analysis was performed with silica plates (Art 5553, Merck, Darmstadt, Germany). Silica gel (0.062–0.2 mm) for column chromatography purification was purchased from Kanto Chemical, Co., Inc. Proton nuclear magnetic resonance (^1^H-NMR) spectra were obtained with JEOL JNM-ECS400 (JEOL Ltd., Tokyo, Japan) and the chemical shifts were reported in ppm units relative to tetramethylsilane (0.0 ppm in CDCl_3_) or residual solvent signal (2.50 ppm in DMSO-d6). Electrospray ionization mass spectra (ESI-MS) were obtained with JEOL JMS-T100TD (JEOL Ltd.). Nanodroplets were prepared from the Bioruptor BR II (BM Equipment Co. Ltd., Tokyo, Japan) and the nanodroplets were extruded through a 100 or 200 nm polycarbonate filter using a LiposoFast manual glass syringe extruder (Avestin Inc., Ottawa, ON, Canada). Particle sizes of nanodroplets were determined by the Nano Partica SZ-100-Z (Horiba, Ltd., Kyoto, Japan). [^111^In]Indium chloride ([^111^In]InCl_3_) (74 MBq/1 mL) was purchased from Nihon Medi-Physics Co., Ltd. (Tokyo, Japan). Purification of radiolabeled nanodroplets was performed on PD-10 column (Cytiva, Westborough, MA, USA). The radioactivity was measured by an Auto Gamma System ARC-7010B (ALOKA Co., Ltd., Tokyo, Japan). Analysis and purification were conducted using an RP-HPLC system (Shimadzu, Kyoto, Japan). A colorectal adenocarcinoma cell line, Colon 26, was obtained from the Cell Resource Centre for Biomedical Research at Tohoku University.

### 4.2. Synthesis of Intermediate Compounds and Precursors

The syntheses of DTPA-TPP (**10**), DTPA-cysteamine-PEG_2000_ (**12**), and their respective intermediate compounds were described as follows.

#### 4.2.1. Synthesis of 5,10,15,20-Tetraphenylporphyrin (TPP) (**1**)

Benzaldehyde (9.3 mL, 91.1 mmol, 1.0 eq.) was dissolved in 150 mL of propionic acid. The mixture was stirred and gradually heated to reflux at 140 °C. During this heating, a solution of pyrrole (7.3 mL, 104.8 mmol, 1.1 eq.) in propionic acid (10 mL) was added dropwise to the reaction mixture over 30–40 min. The mixture was stirred under reflux for 24 h. The reaction progress was monitored by TLC (dichloromethane (DCM)/hexane = 2/1). After the reaction was completed, propionic acid was removed by rotary evaporation. The residue was purified by silica gel column chromatography (eluent: DCM/hexane = 1/5 to 1/0) to afford **1** (1.6 g, 11.4%) as a dark purple solid.

^1^H NMR (400 MHz, CDCl_3_) δ 8.84 (8H, s), 8.23–8.21 (8H, m), 7.79–7.73 (12H, m), −2.78 (2H, s).

#### 4.2.2. Synthesis of 5-(4-Nitrophenyl)-10,15,20-triphenylporphyrin (TPPNO_2_) (**2**)

Compound **1** (797.1 mg, 1.3 mmol, 1.0 eq.) was dissolved in trifluoroacetic acid (TFA) (50 mL). The mixture was cooled to 0 °C by submerging the reaction flask in an ice bath, then sodium nitrate (110 mg, 1.3 mmol, 1.0 eq.) was added. After 3 min of stirring at room temperature, the reaction mixture was diluted with water (200 mL) and extracted with DCM (200 mL), then washed further with saturated aqueous sodium bicarbonate (250 mL). The organic layer was collected and dried over sodium sulfate, filtered, and concentrated by rotary evaporation. The crude product was purified by silica gel column chromatography (eluent: DCM/hexane, 1/2) to afford **2** (192.3 mg, 22.5%) as a dark purple solid.

^1^H NMR (400 MHz, CDCl_3_) δ 8.90 (2H, d, *J* = 4.8 Hz), 8.86 (4H, s), 8.74 (2H, d, *J* = 4.4 Hz), 8.65 (2H, d, *J* = 8.4 Hz), 8.40 (2H, d, *J* = 9.2 Hz), 7.82–7.75 (9H, m), −2.79 (2H, s).

#### 4.2.3. Synthesis of 4-(10,15,20-Triphenylporphyrin-5-yl)aniline (TPPNH_2_) (**3**)

Compound **2** (137.9 mg, 0.2 mmol, 1.0 eq.) was suspended in 12 M aqueous HCl (5 mL) and tin (II) chloride (237.8 mg, 1.3 mmol, 6.0 eq.) was added to the solution. The mixture was heated to 65 °C and stirred for 1 h. The reaction progress was monitored by TLC (DCM/hexane = 1/1). After the reaction was completed, pH of the reaction mixture was adjusted to 9 using 28% ammonia solution, and the mixture was extracted with DCM (2 × 500 mL). The organic layer was collected, dried over sodium sulfate, filtered, and concentrated by rotary evaporation to afford **3** (117.6 mg, 89%) as a dark purple solid.

^1^H NMR (400 MHz, CDCl_3_) δ 8.94 (2H, d, *J* = 4.8 Hz), 8.83 (6H, s), 8.23–8.21 (6H, m), 8.00 (2H, d, *J* = 8.0 Hz), 7.78–7.73 (9H, m), 7.08 (2H, d, *J* = 8.0 Hz), 4.04 (2H, s), −2.76 (2H, s).

#### 4.2.4. Synthesis of (9*H*-Fluoren-9-yl)methyl (1-Oxo-1-((4-(10,15,20-triphenylporphyrin-5-yl)phenyl)amino)-3-(tritylthio)propan-2-yl)-l2-azanecarboxylate (Fmoc-*S*-trityl-l-cysteine-TPP) (**4**)

To a solution of **3** (114.8 mg, 0.2 mmol, 1.0 eq.) dissolved in anhydrous dimethylformamide (DMF) (2 mL), *N*-Fmoc-*S*-trityl-l-cysteine (117.4 mg, 0.2 mmol, 1.1 eq.), HOAt (27.3 mg, 0.2 mmol, 1.1 eq.), HATU (76.2 mg, 0.2 mmol, 1.1 eq.), and DIPEA (63.5 µL, 0.4 mmol, 2.0 eq.) were added successively. The resulting mixture was stirred at room temperature for 1 h. The reaction progress was monitored by TLC (DCM/hexane = 10/1). After the reaction was completed, saturated aqueous sodium bicarbonate (150 mL) was added to the reaction mixture and the mixture was extracted with DCM (150 mL). The organic phase was separated, washed with water (100 mL), dried over sodium sulfate, filtered, and concentrated by rotary evaporation. The crude product was purified by silica gel column chromatography (eluent: DCM/hexane, 10/1) to afford **4** (204 mg, 94%) as a dark purple solid.

^1^H NMR (400 MHz, CDCl_3_) δ 8.84 (8H, s), 8.21 (6H, d, *J* = 6.8 Hz), 8.15 (2H, d, *J* = 8.4 Hz), 7.83 (2H, d, *J* = 8.4 Hz), 7.80–7.73 (11H, m), 7.63 (2H, d, *J* = 7.6 Hz), 7.52 (6H, d, *J* = 8.0 Hz), 7.44–7.38 (2H, m), 7.36–7.27 (11H, m), 5.08–5.02 (1H, m), 4.54 (2H, d, *J* = 6.4 Hz), 4.27 (1H, t, *J* = 6.8 Hz), 4.01–3.96 (1H, m), 2.95–2.84 (2H, m), −2.80 (2H, s).

#### 4.2.5. Synthesis of 2-Amino-*N*-(4-(10,15,20-triphenylporphyrin-5-yl)phenyl)-3-(tritylthio)propenamide (*S*-Trityl-l-cysteine-TPP) (**5**)

Compound **4** (188.4 mg, 0.2 mmol) was dissolved in a mixture of DMF (2.4 mL) and piperidine (0.6 mL). The reaction mixture was stirred at room temperature for 1 h. The reaction progress was monitored by TLC (hexane/ethyl acetate = 1/1). After the reaction was completed, the reaction mixture was concentrated by rotary evaporation, and the resulting residue was subjected to a phase separation with saturated aqueous sodium bicarbonate (50 mL) and ethyl acetate (50 mL). The organic layer was collected, dried over sodium sulfate, filtered, and concentrated by rotary evaporation. The crude product was purified by silica gel column chromatography (eluent: hexane/ethyl acetate, 5/1 to 2/1) to afford **5** (140.1 mg, 91%) as a dark purple solid.

^1^H NMR (400 MHz, CDCl_3_) δ 9.65 (1H, s), 8.85–8.84 (8H, m), 8.22–8.20 (6H, m), 8.15 (2H, d, *J* = 8.8 Hz), 7.93 (2H, d, *J* = 8.4 Hz), 7.80–7.72 (9H, m), 7.55–7.53 (6H, m), 7.37–7.25 (9H, m), 3.34–3.31 (1H, m), 3.01–2.81 (2H, m), −2.79 (2H, s).

#### 4.2.6. Synthesis of 2,5-Dioxopyrrolidin-1-yl 6-((((9*H*-fluoren-9-yl)methoxy)carbonyl)amino)hexanoate (Fmoc-6-Ahx-NHS) (**6**)

To a solution of 6-(Fmoc-amino)hexanoic acid (157.9 mg, 0.4 mmol, 1.0 eq.) and NHS (61.7 mg, 0.5 mmol, 1.2 eq.) in DCM (8 mL), EDCI hydrochloride (EDCI·HCl) (102.8 mg, 0.5 mmol, 1.2 eq.) was added. The mixture was stirred for 5 h at room temperature. The reaction progress was monitored by TLC (hexane/ethyl acetate = 1/1). After the reaction was completed, the mixture was concentrated by rotary evaporation, and the residue was purified by silica gel column chromatography (eluent: hexane/ethyl acetate, 2/1) to afford **6** (163.5 mg, 81%) as a colorless solid.

^1^H NMR (400 MHz, CDCl_3_) δ 7.77 (2H, d, *J* = 7.6 Hz), 7.61 (2H, d, *J* = 7.6 Hz), 7.42–7.39 (2H, t, *J* = 7.4 Hz), 7.34–7.30 (2H, t, *J* = 8.0 Hz), 4.93 (1H, bs), 4.40 (2H, d, *J* = 6.8 Hz), 4.24–4.20 (1H, m), 3.25–3.20 (2H, m), 2.83 (4H, bs), 2.65–2.61 (2H, t, *J* = 7.0 Hz), 1.81–1.77 (2H, m), 1.48–1.44 (2H, m).

#### 4.2.7. Synthesis of (9*H*-Fluoren-9-yl)methyl (6-oxo-6-((1-oxo-1-((4-(10,15,20-triphenylporphyrin-5-yl)phenyl)amino)-3-(tritylthio)propan-2-yl)amino)hexyl)carbamate (Fmoc-6-Ahx-*S*-trityl-l-cysteine-TPP) (**7**)

To a solution of compound **5** (142.1 mg, 0.15 mmol, 1.0 eq.) in anhydrous DMF (1 mL), compound **6** (98.5 mg, 0.23 mmol, 1.5 eq.) was added at room temperature. The mixture was stirred for 3 h at 40 °C. The reaction progress was monitored by TLC (hexane/ethyl acetate = 1/1). After the reaction was completed, the mixture was concentrated by rotary evaporation, and the residue was purified by silica gel column chromatography (eluent: hexane/ethyl acetate, 2/1 to 1/1) to afford **7** (84.2 mg, 44%) as a dark purple solid.

^1^H NMR (400 MHz, CDCl_3_) δ 8.83–8.59 (8H, m), 8.22–8.19 (6H, m), 8.13 (2H, d, *J* = 7.6 Hz), 7.84 (2H, d, *J* = 8.4 Hz), 7.78–7.72 (9H, m), 7.69 (2H, d, *J* = 7.2 Hz), 7.55–7.52 (8H, m), 7.38–7.28 (13H, m), 5.86–5.82 (1H, m), 4.84–4.80 (1H, m), 4.38–4.37 (2H, m), 4.35–4.31 (1H, m), 4.18–4.17 (1H, m), 3.23–3.18 (2H, m), 3.02–2.79 (2H, m), 2.24–2.20 (2H, m), 1.73–1.69 (2H, m), 1.44–1.38 (2H, m), −2.80 (2H, s).

#### 4.2.8. Synthesis of 5-Amino-*N*-(1-oxo-1-((4-(10,15,20-triphenylporphyrin-5-yl)phenyl)amino)-3-(tritylthio)propan-2-yl)pentanamide (6-Ahx-*S*-trityl-l-cysteine-TPP) (**8**)

Compound **7** (32.9 mg, 25.1 µmol) was dissolved in a mixture of DMF (0.5 mL) and piperidine (0.1 mL). The reaction mixture was stirred at room temperature for 1 h. The reaction progress was monitored by TLC (chloroform/methanol = 9/1). After the reaction was completed, the reaction mixture was concentrated by rotary evaporation, and the resulting residue was purified by silica gel column chromatography (eluent: chloroform/methanol, 9/1) to afford **8** (15.7 mg, 58%) as a dark purple solid.

^1^H NMR (400 MHz, CDCl_3_) δ 8.83–8.69 (8H, m), 8.21–8.20 (6H, m), 8.14 (2H, d, *J* = 8.4 Hz), 7.85 (2H, d, *J* = 8.8 Hz), 7.80–7.73 (9H, m), 7.55–7.52 (6H, m), 7.39–7.28 (9H, m), 6.08–6.06 (1H, m), 4.36–4.34 (1H, m), 3.02–2.81 (2H, m), 2.74–2.71 (2H, t, *J* = 7.0 Hz), 2.26–2.22 (2H, t, *J* = 7.4 Hz), 1.72–1.67 (2H, m), 1.43–1.39 (2H, m), −2.80 (2H, s).

#### 4.2.9. Synthesis of 2,2′-((2-((2-(Bis(carboxymethyl)amino)-3-(4-(3-(5-oxo-5-((1-oxo-1-((4-(10,15,20-triphenylporphyrin-5-yl)phenyl)amino)-3-(tritylthio)propan-2-yl)amino)pentyl)thioureido)phenyl)propyl)(carboxymethyl)amino)ethyl)azanediyl)diacetic acid (DTPA-6-Ahx-*S*-trityl-l-cysteine-TPP) (**9**)

To a solution of **8** (15.7 mg, 14.4 µmol, 1.0 eq.) and *S*-2-(4-isothiocyanatobenzyl)-diethylenetriamine pentaacetic acid (*p*-SCN-Bn-DTPA) (9.4 mg, 14.5 µmol, 1.0 eq.) in 0.5 mL anhydrous DMF, DIPEA (50.3 µL, 0.3 mmol, 20.0 eq) was added. The mixture was stirred at room temperature for 1 h under a nitrogen atmosphere. The reaction progress was monitored by TLC (chloroform/methanol = 2/1). After the reaction was completed, the reaction mixture was concentrated by rotary evaporation, and the resulting residue was subjected to a phase separation with water (50 mL) and chloroform (50 mL). The organic layer was collected, dried over sodium sulfate, filtered, and concentrated by rotary evaporation. The crude material was purified by RP-HPLC using Cosmosil 5C_18_-MS-II (10ID × 150 mm) column at a flow rate of 4 mL/min, column temperature maintained at 40 °C, with a gradient mobile phase of 40% acetonitrile in water with 0.1% triethylamine (TEA) to 60% acetonitrile in water with 0.1% TEA for 20 min, to afford **9** (9.5 mg, 66%) as a purple solid.

LRMS (ESI+) calcd for C_94_H_87_N_11_NaO_12_S_2_ [M+Na]+: *m*/*z* = 1651 found 1651.

#### 4.2.10. Synthesis of 2,2′-((2-((2-(Bis(carboxymethyl)amino)-3-(4-(3-(5-((3-mercapto-1-oxo-1-((4-(10,15,20-triphenylporphyrin-5-yl)phenyl)amino)propan-2-yl)amino)-5-oxopentyl)thioureido)phenyl)propyl)(carboxymethyl)amino)ethyl)azanediyl)diacetic acid (DTPA-TPP) (**10**)

Compound **9** (9.5 mg, 5.8 µmol) was treated with a mixture of 95% TFA, 2.5% triisopropylsilane (TIS) and 2.5% water for 1 h at room temperature. After the reaction was completed, the crude sample was washed with a mixture of hexane/ethyl acetate (5/1) to obtain **10** (7.7 mg, 95%) as a dark green solid. The obtained compound was used directly for nanodroplet conjugation without further purification.

LRMS (ESI+) calcd for C_75_H_76_N_11_O_12_S_2_ [M+H]+: *m*/*z* = 1387 found 1387.

#### 4.2.11. Synthesis of 2,2′-((2-((2-(Bis(carboxymethyl)amino)-3-(4-(3-(2-mercaptoethyl)thioureido)phenyl)propyl)(carboxymethyl)amino)ethyl)azanediyl)diacetic acid (DTPA-cysteamine) (**11**)

To a solution of *p*-SCN-Bn-DTPA (24.5 mg, 37.7 µmol, 1.0 eq.) and cysteamine hydrochloride (11.3 mg, 0.1 mmol, 3.0 eq.) in 0.5 mL anhydrous DMF, TEA (78.8 µL, 0.6 mmol, 15.0 eq.) was added. The mixture was stirred at room temperature for 1 h under nitrogen gas. After the reaction was completed, the crude material was purified by RP-HPLC using Cosmosil 5C_18_-AR-II (10ID × 150 mm) column at a flow rate of 4 mL/min, column temperature maintained at 40 °C, with a gradient mobile phase of 15% methanol in water with 0.1% TFA to 30% methanol in water with 0.1% TFA for 15 min, to afford **11** (21.0 mg, 66%) as a white solid.

LRMS (ESI+) calcd for C_24_H_36_N_5_O_10_S_2_ [M+H]+: *m*/*z* = 618 found 618.

#### 4.2.12. Synthesis of 1,2-Distearoyl-*sn*-glycero-3-phosphoethanolamine-*N*-[maleimide(polyethylene Glycol)-2000]-2,2′-((2-((2-(bis(carboxymethyl)amino)-3-(4-(3-(2-mercaptoethyl)thioureido)phenyl)propyl)(carboxymethyl)amino)ethyl)azanediyl)diacetic acid (DTPA-cysteamine-PEG_2000_) (**12**)

To a solution of *N*-[(3-maleimide-1-oxopropyl)aminopropyl polyethyleneglycol-carbamyl] distearoylphosphatidyl-ethanolamine (DSPE-PEG_2000_-maleimide) (7.2 mg, 5.8 µmol, 1.0 eq.) and **11** (17.1 mg, 11.7 µmol, 2.0 eq.) in 0.5 mL of 0.1 M phosphate buffer containing 0.15 M sodium chloride and 2 mM ethylenediaminetetraacetic acid (EDTA), TEA (1.6 µL, 11.7 mmol, 2.0 eq.) was added. The mixture was stirred at room temperature for 24 h. After the reaction was completed, the crude material was purified by manual dialysis. 1 mL of the crude mixture was filled into a dialysis tubing (1 kDa), then dialyzed in 1 L of Milli-Q water. The surrounding solvent was replaced thrice every 12 h with a new 1 L of Milli-Q water. Compound **12** was recovered as a white solid (15.5 mg, 75%). A comparative ^1^H NMR between the starting material, DSPE-PEG_2000_-maleimide, and **12** was conducted for product confirmation. The absence of 2H from the maleimide group of DSPE-PEG_2000_-maleimide at 6.9 ppm indicated that conjugation had occurred. **12** was used directly for nanodroplet synthesis.

### 4.3. Synthesis of Nanodroplets

Nanodroplets were prepared by the thin film hydration technique as previously described [35]. The lipids, DMPA, DPPC, cholesterol, 1,2-distearoyl-*sn*-glycero-3-phosphoethanolamine-*N*-[methoxy(polyethylene glycol)-2000], DSPE-PEG_2000_-maleimide, and IR780 were dissolved in chloroform at a molar ratio of 9.5:38:47.5:4:1:0.1. Chloroform was removed from the lipid solution by rotary evaporation, and the residual solvent was dried further in a desiccator under vacuum for 1 h. The dried thin film was hydrated with 6 mL of PBS without calcium chloride or magnesium chloride to achieve a total lipid concentration of 4 mM. The hydrated lipid film was then briefly sonicated to facilitate detachment and dispersion of the lipid layer into the aqueous phase. Once the suspension appeared homogeneous, 0.3 mL of PFH was added to the sample. An emulsion was made by sonicating the sample on ice using a 20 kHz ultrasonic processor at an emission power of 935 W, for 10 cycles with a total duration of 5 min (30 s for each sonication with a 30 s interval between each cycle). The emulsion was then sequentially extruded 21 times through a 200 nm polycarbonate filter using a glass syringe (0.5 mL) hand extruder. For the surface conjugation of porphyrin derivative, 1 eq. of DSPE-PEG_2000_-maleimide in the nanodroplets was reacted with 1 eq. of DTPA-TPP (**10**), accompanied by stirring at room temperature for 2 h. Dynamic light scattering was used to determine particle sizes of the nanodroplets.

### 4.4. Radiolabeling of Nanodroplets

[^111^In]In-DTPA-cysteamine-nanodroplets and [^111^In]In-DTPA-TPP-nanodroplets were synthesized by a simple chelation reaction of [^111^In]InCl_3_ with DTPA moiety of nanodroplets. To a nanodroplet solution containing 3 μmol of total lipid, 20 µL of 1 M acetate buffer (pH 4.5) was first added, followed by [^111^In]InCl_3_ (20–200 MBq/mL). The resulting reaction mixture with a total volume of 0.3–0.4 mL was incubated at 37 °C for 1 h. After the reaction was complete, the radiolabeled nanodroplets were purified using a PD-10 column, eluted with PBS. Radiochemical yield and purity were determined by an auto well gamma counter.

### 4.5. Cellular Uptake Experiments

Cellular uptake experiments were performed according to a previously reported method [36]. Colon 26 cells were cultured in RPMI-1640 medium supplemented with 10% fetal bovine serum (FBS) on 6 well culture plates (3 × 10^5^ cells/2 mL/well). After 24 h incubation, the medium was removed and a solution of [^111^In]In-DTPA-cysteamine-nanodroplets or [^111^In]In-DTPA-TPP-nanodroplets (37 kBq/well) in 2 mL medium with or without FBS was added and incubated for 1, 3, and 6 h. For the blocking study, free TPP (10 µM) was also added to a solution of [^111^In]In-DTPA-TPP-nanodroplets (37 kBq/well) in medium with FBS and incubated for 1 h. Once incubation time points were reached, the medium from each well was removed. Cells were washed twice with ice-cold PBS (1 mL) and then lysed twice in 0.5 mL of 1 M NaOH aqueous solution. The radioactivity was determined by an auto-well gamma counter. The total cell protein was quantified with a BCA protein assay kit (Nacalai Tesque, Inc.) following the manufacturer’s protocol. All data were expressed as percent dose per milligram protein (%dose/mg protein).

### 4.6. Animals

All animal handling procedures were approved by the Kanazawa University Animal Care Committee. Experiments with animals were conducted in accordance with the Guidelines for the Care and Use of Laboratory Animals of Kanazawa University. The animals were housed with free access to food and water at 23 °C with a 12 h (light/dark) schedule. Six-week-old male ddY (27–30 g) and four-week-old BALB/c (17–19 g) were purchased from Japan SLC Inc. (Hamamatsu, Japan).

#### 4.6.1. Biodistribution Study

The biodistribution of [^111^In]In-DTPA-cysteamine-nanodroplets and [^111^In]In-DTPA-TPP-nanodroplets was evaluated in five-week-old female BALB/c mice. The biodistribution of [^111^In]InCl_3_ was evaluated in six-week-old male ddY mice. Mice were inoculated with Colon 26 cells (5 × 10^6^ cells) in 100 µL of PBS by subcutaneous injection into the right shoulder. Once tumors reached a palpable size, a PBS solution of [^111^In]In-DTPA-cysteamine-nanodroplets or [^111^In]In-DTPA-TPP-nanodroplets (37 kBq, 100 µL) was injected intravenously into the tail vein of the tumor-bearing mice. Mice were sacrificed at 1, 6, 24, and 48 h postinjection. Tissues of interest were excised and weighed, their radioactivity was measured by an auto-well gamma counter, and the counts were corrected for background radiation.

#### 4.6.2. SPECT/CT Imaging

SPECT/CT imaging of [^111^In]In-DTPA-TPP-nanodroplets in Colon 26 tumor-bearing mice was performed using a small animal SPECT system (VECTor/CT, MIlabs, Houten, The Netherlands). SPECT scanning was performed for 30 min from 24 h postinjection of [^111^In]In-DTPA-TPP-nanodroplets (7.4 MBq). Data were acquired in list mode, and photopeak windows were set after the acquisition. The energy windows of 156–190 and 222–272 keV were employed. Data were reconstructed using pixel-based order-subsets expectation maximization, with correction for attenuation on computed tomography, in 16 subsets and 6 iterations. The voxel size was 0.8 × 0.8 × 0.8 mm. The obtained SPECT/CT images were analyzed using an image-processing application (AMIDE Imaging software, version 1.0.4).

#### 4.6.3. In Vivo SDT Assessment

SDT was performed according to previous reports with slight modifications [37]. Female BALB/c mice (5 weeks) were inoculated with Colon 26 cells (1 × 10^6^ cells) in 100 µL of PBS by subcutaneous injection into the right and left shoulders, respectively. Once tumor sizes reached 50–100 mm^3^, a PBS solution of DTPA-TPP-nanodroplets at 10 µmol of total lipids/kg of mice body weight was injected intravenously. For SDT, an ultrasound transducer at a frequency of 1 MHz, with an intensity of 2.4 W/cm^2^ and a 50% duty cycle, was utilized. Mice were anesthetized prior to ultrasound. At 6 h postinjection, ultrasound was irradiated on the left shoulder tumor for a duration of 3 min while the right shoulder tumor was left untreated. Mice were monitored for tumor growth and body weight up to 7 days postinjection. Tumor size was measured with a digital slide caliper, and tumor volume was calculated as volume (V) = [length × width^2^]/2 and compared to the values of day 0 (relative tumor volume). On day 7 postinjection, the mice were sacrificed, tumors on both shoulders were dissected, and their weights were measured.

### 4.7. Statistical Analysis

All data were analyzed using GraphPad Prism 5.04 (GraphPad Software Inc., San Diego, CA, USA) and displayed as mean ± standard deviation (SD). The significant difference in cellular uptake and biodistribution experiments between [^111^In]In-DTPA-TPP-nanodroplets and [^111^In]In-DTPA-cysteamine-nanodroplets was determined using an unpaired Student’s *t*-test. Significant differences in relative tumor volume and tumor weights among control groups and DTPA-TPP-nanodroplet treated groups were calculated by one-way analysis of variance (ANOVA) followed by Tukey’s post hoc test.

## 5. Conclusions

The developed ^111^In-labeled nanodroplets, [^111^In]In-DTPA-TPP nanodroplets, and their nonradioactive derivative, DTPA-TPP nanodroplets, are excellent candidates as theranostic agents. Compared with control nanodroplets without TPP, [^111^In]In-DTPA-TPP nanodroplets had higher cellular uptake. This observation was consistent with the biodistribution profile, which showed higher tumor accumulation of [^111^In]In-DTPA-TPP nanodroplets. Additionally, the tumor accumulation was visualized using SPECT imaging. Furthermore, the in vivo SDT results revealed remarkable antitumor effects of DTPA-TPP nanodroplets. These results indicate the applicability of combining SPECT imaging and SDT with nanodroplets for prospective cancer theranostics.

## Figures and Tables

**Table 1:** Biodistribution of [^111^In]In-DTPA-cysteamine and [^111^In]In-DTPA-TPP nanodroplets at 1, 6, 24, and 48 h after intravenous injection in Colon 26 tumor-bearing mice.

Tissues	Time After Injection			
	1 h	6 h	24 h	48 h
[^111^In]In-DTPA-cysteamine nanodroplets				
Blood	13.87 (4.62)	8.21 (1.41)	0.87 (0.21)	0.17 (0.01)
Liver	50.15 (5.07)	34.86 (2.38)	35.69 (3.42)	26.06 (0.60)
Kidneys	5.97 (0.80)	6.40 (0.55)	4.15 (0.70)	2.68 (0.13)
Small Intestine	4.26 (1.19)	1.42 (0.12)	1.27 (0.23)	0.83 (0.06)
Large Intestine	1.60 (1.20)	2.97 (0.83)	0.84 (0.12)	0.47 (0.04)
Spleen	63.53 (8.76)	53.49 (3.79)	43.74 (5.14)	24.70 (1.92)
Pancreas	1.03 (0.64)	0.70 (0.06)	0.35 (0.08)	0.34 (0.26)
Lung	11.30 (1.79)	5.13 (1.19)	1.48 (0.34)	0.69 (0.08)
Stomach	0.24 (0.05)	0.15 (0.02)	0.11 (0.02)	0.06 (0.01)
Heart	2.04 (0.38)	1.61 (0.23)	0.65 (0.12)	0.38 (0.06)
Muscle	0.62 (0.18)	0.43 (0.02)	0.28 (0.06)	0.21 (0.07)
Brain	0.27 (0.06)	0.21 (0.04)	0.04 (0.01)	0.02 (0.00)
Bone	3.95 (0.71)	2.95 (0.08)	2.39 (0.44)	1.81 (0.20)
Tumor	1.20 (0.08)	2.15 (0.38)	1.83 (0.32)	1.34 (0.26)
Tumor/blood	0.09 (0.03)	0.26 (0.04)	2.16 (0.37)	8.06 (1.41)
Tumor/muscle	2.05 (0.49)	4.95 (0.80)	6.67 (0.76)	6.91 (1.82)
[^111^In]In-DTPA-TPP nanodroplets				
Blood	15.30 (2.98)	5.23 (0.51) *	0.51 (0.16)	0.17 (0.02)
Liver	65.54 (3.62) **	72.06 (2.91) ***	44.80 (9.30) **	34.12 (2.03) ***
Kidneys	3.06 (0.36) **	2.39 (0.11) ***	1.54 (0.02) **	1.03 (0.04) ***
Small Intestine	1.39 (0.18) **	1.44 (0.11)	1.35 (0.29)	0.62 (0.04) **
Large Intestine	0.37 (0.07)	2.98 (0.23)	2.18 (0.27) ***	0.90 (0.08) ***
Spleen	49.65 (16.69)	40.21 (3.53) **	35.99 (10.56) *	20.65 (2.60)
Pancreas	0.55 (0.16)	0.42 (0.05) ***	0.29 (0.03)	0.24 (0.02)
Lung	7.85 (0.87) *	3.76 (0.05)	1.60 (0.10)	0.80 (0.20)
Stomach	0.15 (0.06) *	0.18 (0.12)	0.60 (0.10)	0.29 (0.07)
Heart	2.04 (0.30)	1.30 (0.14)	0.49 (0.09)	0.36 (0.03)
Muscle	0.36 (0.02)	0.34 (0.07) *	0.19 (0.01)	0.13 (0.02)
Brain	0.26 (0.06)	0.11 (0.01)	0.04 (0.00)	0.02 (0.01)
Bone	2.22 (0.65) *	1.98 (0.19) ***	1.47 (0.13) *	0.81 (0.19) **
Tumor	1.62 (0.23) *	3.39 (0.30) **	2.76 (0.30) *	1.56 (0.22)
Tumor/blood	0.11 (0.03)	0.65 (0.04) ***	5.85 (2.24) *	9.24 (1.39)
Tumor/muscle	4.45 (0.79)	10.16 (1.27) **	14.79 (0.76) ***	11.91 (3.05)

Data presented as % injected dose/gram tissue. Each value represents mean ± SD for four animals. Significance was determined using unpaired Student’s *t*-test (* *p* < 0.05, ** *p* < 0.01, *** *p* < 0.001 vs. [^111^In]In-DTPA-cysteamine nanodroplets).

## Data Availability

The original contributions presented in the study are included in the article/Supplementary Material; further inquiries can be directed to the corresponding author.

## Supplementary Materials

The following supporting information can be downloaded at https://www.mdpi.com/article/10.3390/molecules31071114/s1. ^1^H NMR spectra of **1**–**8**, and **12**; MS (ESI+) spectra of **9**–**11**; Figure S1: HPLC chromatograms of **9** and **10**; Figure S2: Particle size distribution; Figure S3: Cellular uptake; Figure S4: Body weight of mice; Table S1: Biodistribution of [^111^In]InCl_3_; Table S2: Detailed SDT data.

## Abbreviations

The following abbreviations are used in this manuscript:

EPR	Enhanced Permeability and Retention
PDT	Photodynamic Therapy
ROS	Reactive Oxygen Species
PFC	Perfluorocarbon
PFH	Perfluorohexane
SDT	Sonodynamic Therapy
^111^In	Indium-111
PEG	Polyethylene Glycol
SPECT	Single Photon Emission Computed Tomography
^1^H-NMR	Proton Nuclear Magnetic Resonance
ESI-MS	Electrospray Ionization Mass Spectra
InCl_3_	Indium Chloride
TPP	5,10,15,20-Tetraphenylporphyrin
TFA	Trifluoroacetic Acid
DMF	Dimethylformamide
HATU	Hexafluorophosphate Azabenzotriazole Tetramethyl Uranium
DIPEA	*N*,*N*-Diisopropylethylamine
DCM	Dichloromethane
NHS	*N*-Hydroxysuccinimide
EDCI·HCl	1-Ethyl-3-(3-dimethylaminopropyl)carbodiimide Hydrochloride
*p*-SCN-Bn-DTPA	*S*-2-(4-Isothiocyanatobenzyl)-diethylenetriamine Pentaacetic Acid
EDTA	Ethylenediaminetetraacetic Acid
DSPE-PEG_2000_-Maleimide	*N*-[(3-Maleimide-1-oxopropyl)aminopropyl Polyethyleneglycol-carbamyl] distearoylphosphatidyl-ethanolamine
IR780iodide	(2-[2-[2-Chloro-3-[(1,3-dihydro-3,3-dimethyl-1-propyl-2*H*-indol-2-ylidene)ethylidene]-1-cyclohexen-1-yl]ethenyl]-3,3-dimethyl-1-propylindolium iodide)
PBS	Phosphate Buffered Saline
SD	Standard Deviation
ANOVA	One-Way Analysis of Variance
