# Supplementary material for: Preparation and Evaluation of Radiolabeled Porphyrin-Functionalized Lipid Nanodroplets for Cancer Theranostics
Source: Molecules. 2026 Mar 27;31(7):1114. doi: 10.3390/molecules31071114 (PMC13074605; doi:10.3390/molecules31071114)
Supplement: Supplementary file 1 [file molecules-31-01114-s001.zip › molecules-4181030-supplementary.pdf]

Supplementary data

Preparation and Evaluation of Radiolabeled Porphyrin-Functionalized Nanodroplets for Cancer Theranostics

Nur Izni Binti Ramzi[1,†], Kisa Tamamura[1,†], Masayuki Munekane[1,†], Kenji Mishiro[1], Takeshi Fuchigami[1], Xiaojun Hu[2], Renata Jastrząb[3], Seigo Kinuya[4], Kazuaki Ninomiya[5], Kazuma Ogawa[1,*]

[1]*Graduate School of Medical Sciences, Kanazawa University, Kakuma-machi, Kanazawa 920-1192, Ishikawa, Japan;*

[2]*Center for Molecular Recognition and Biosensing, School of Life Sciences, Shanghai University, Shanghai 200444, China;*

[3]*Faculty of Chemistry, Adama Mickiewicz University in Poznan, Uniwersytetu Poznańskiego 8, Poznan 61-614, Poland;*

[4]*Department of Nuclear Medicine, Kanazawa University Hospital, Kanazawa University, Takara-machi 13-1, Kanazawa 920-8641, Ishikawa, Japan;*

[5] *Faculty of Biological Science and Technology, Institute of Science and Engineering, Kanazawa University, Kakuma-machi, Kanazawa 920-1192, Ishikawa, Japan.*

[†]These three authors contributed equally to this work.

*Corresponding Author

Kazuma Ogawa

Graduate School of Medical Sciences, Kanazawa University, Kakuma-machi, Kanazawa 920-1192; Japan.

Telephone: 81-76-234-4460; Fax: 81-76-234-4460

E-mail: kogawa@p.kanazawa-u.ac.jp

<u>**Table of Contents**</u>

1. ^{1}H NMR spectra of 1-8 .... S3-S10

2. MS (ESI+) spectra of 9-11 .... S11-12

3. Figure S1. HPLC chromatograms of 9 and 10 .... S13

4. Figure S2. Particle size distribution S14

5. Figure S3. Cellular uptake S15

6. Table S1. Biodistribution S16

7. Table S2. Detailed SDT data S17

8. Figure S4. Body weight of mice S18

1. ^{1}H NMR spectra of intermediates and precursors compounds (1-8) of L-cysteine-DTPA-TPP (10)

^{1}H NMR spectrum of **1** (400 MHz, $CDCl_3$)

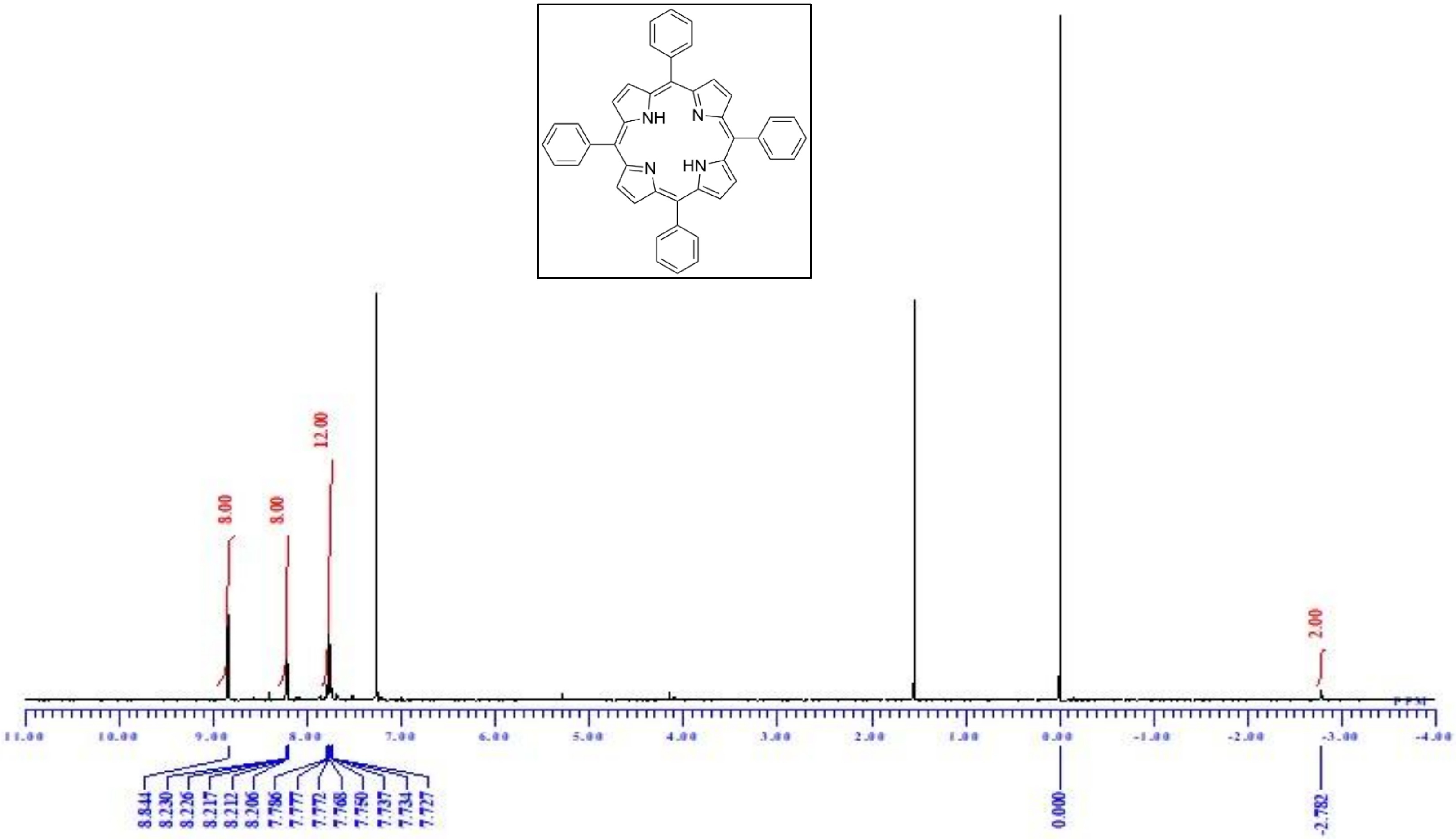

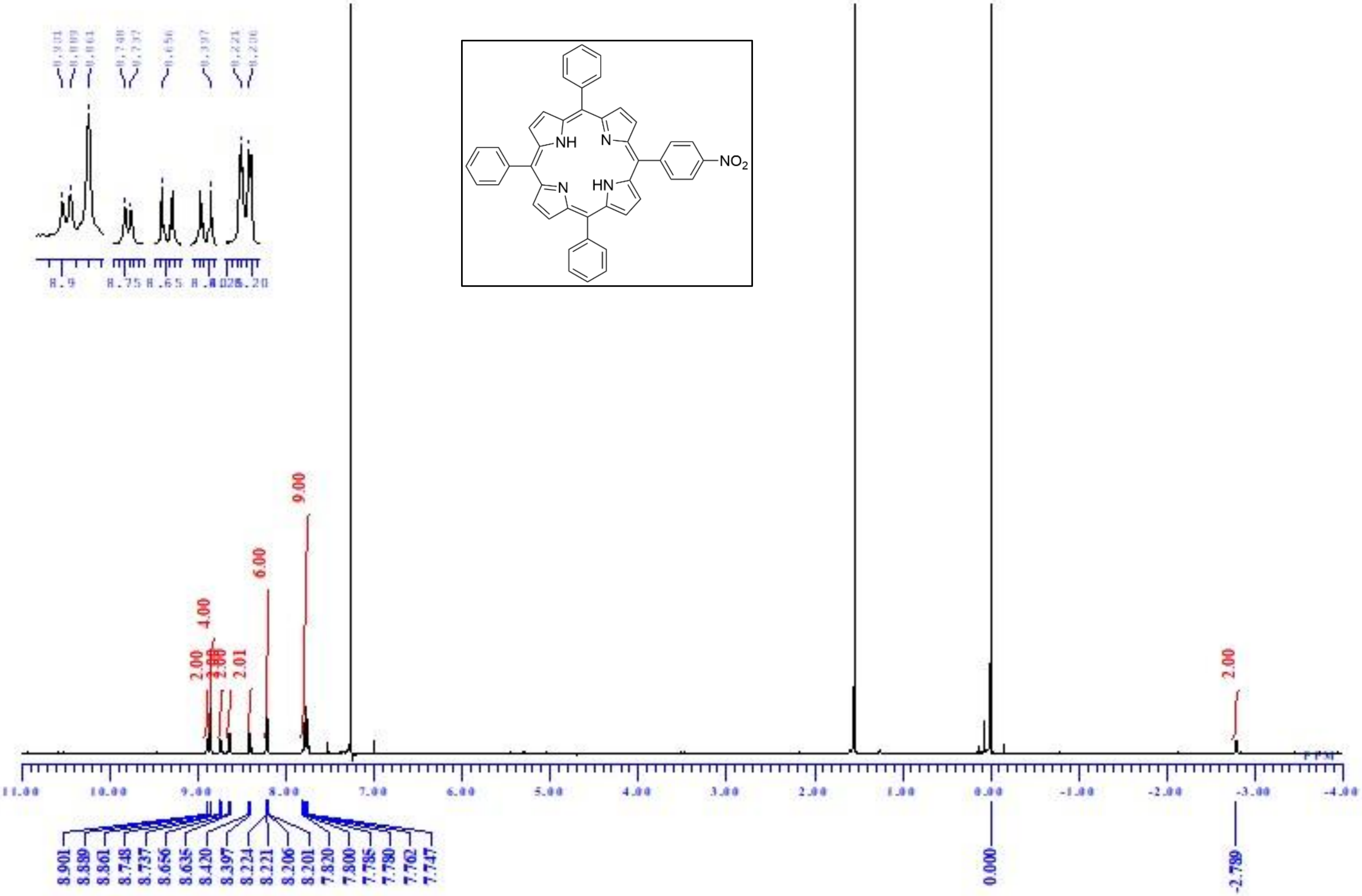

^{1}H NMR spectrum of **2** (400 MHz, $CDCl_3$)

^{1}H NMR spectrum of **3** (400 MHz, $CDCl_3$)

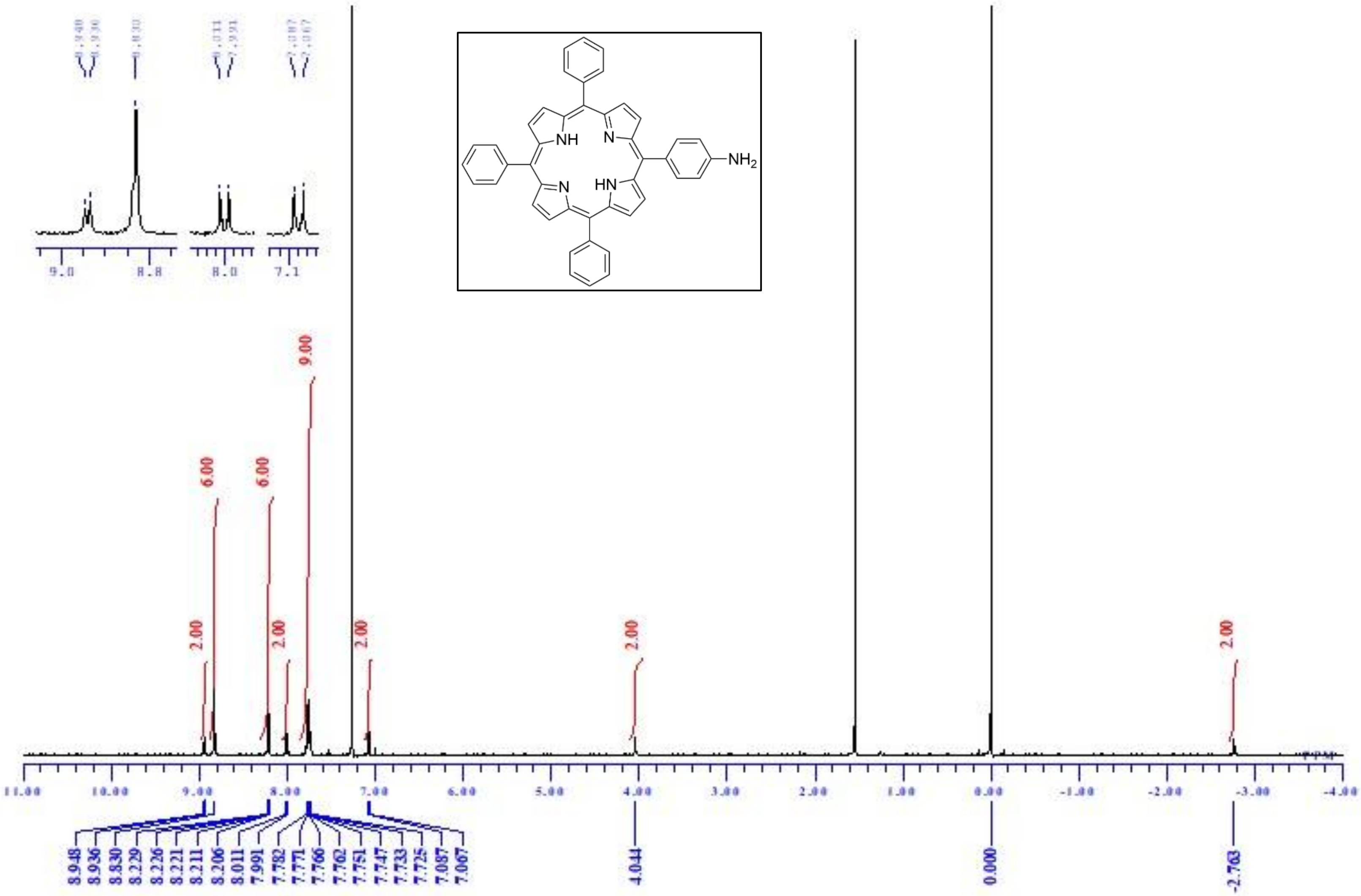

NMR spectrum of **4** (400 MHz, $CDCl_3$)

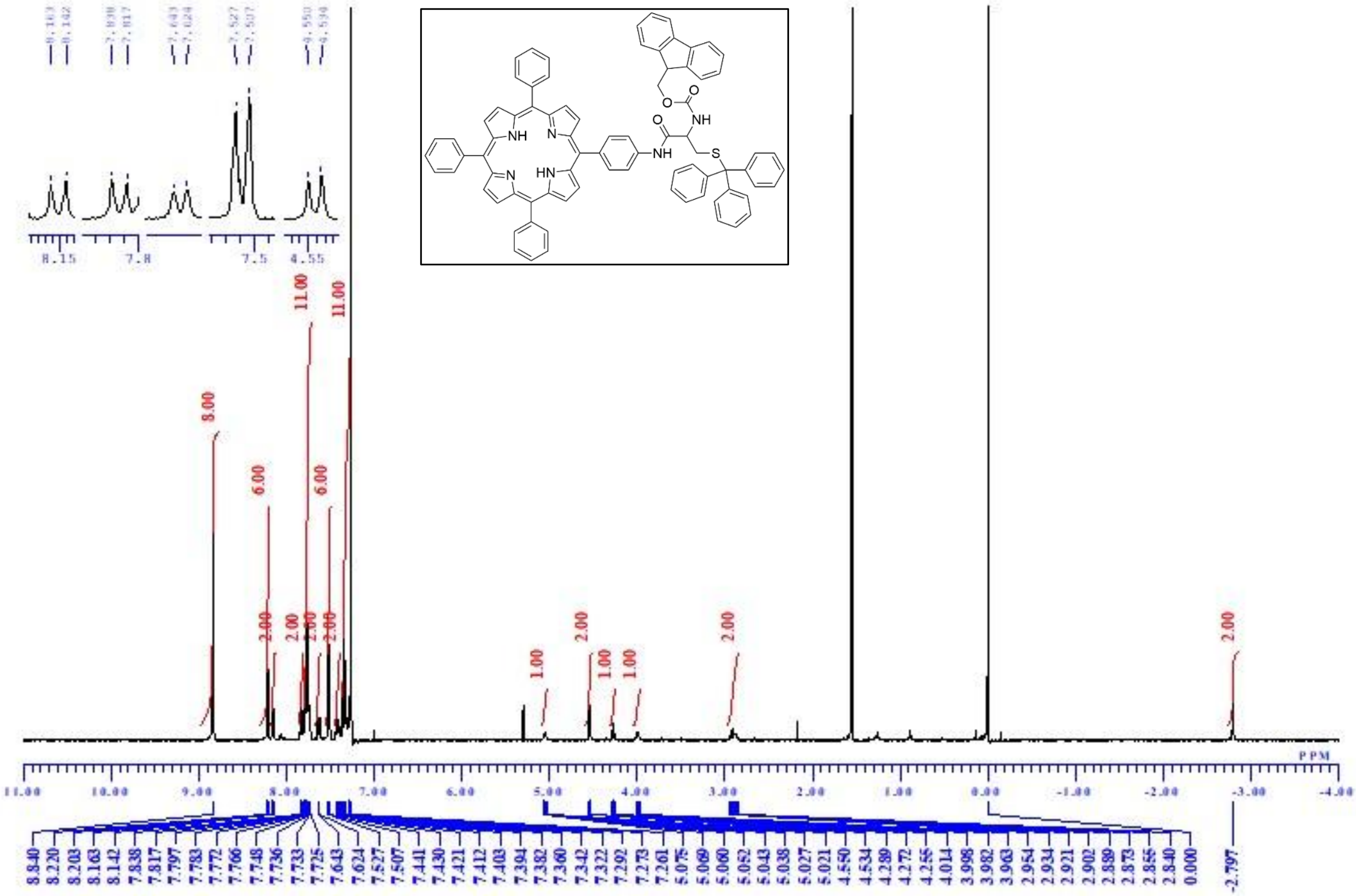

NMR spectrum of **5** (400 MHz, $CDCl_3$)

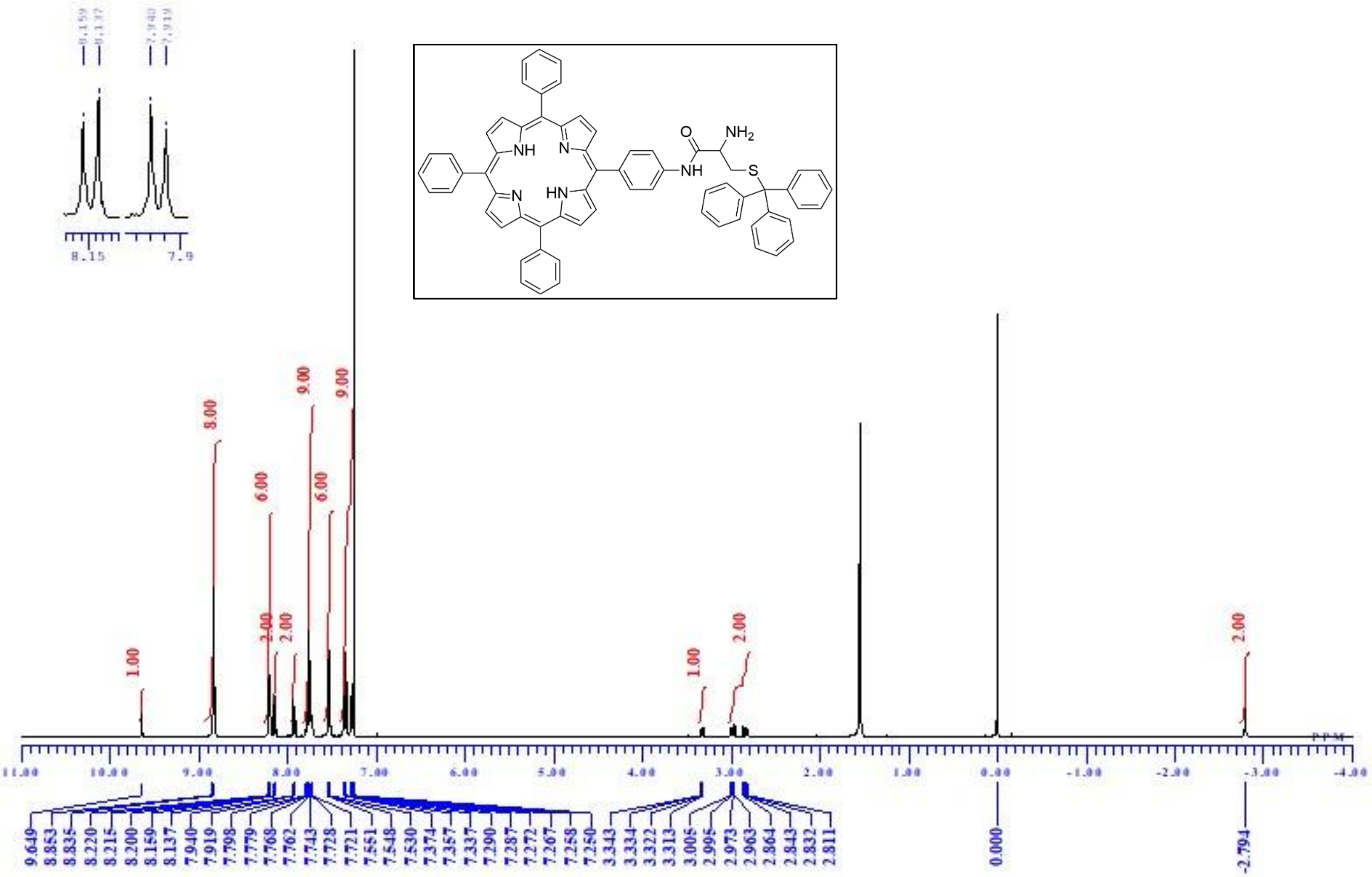

NMR spectrum of **6** (400 MHz, $CDCl_3$)

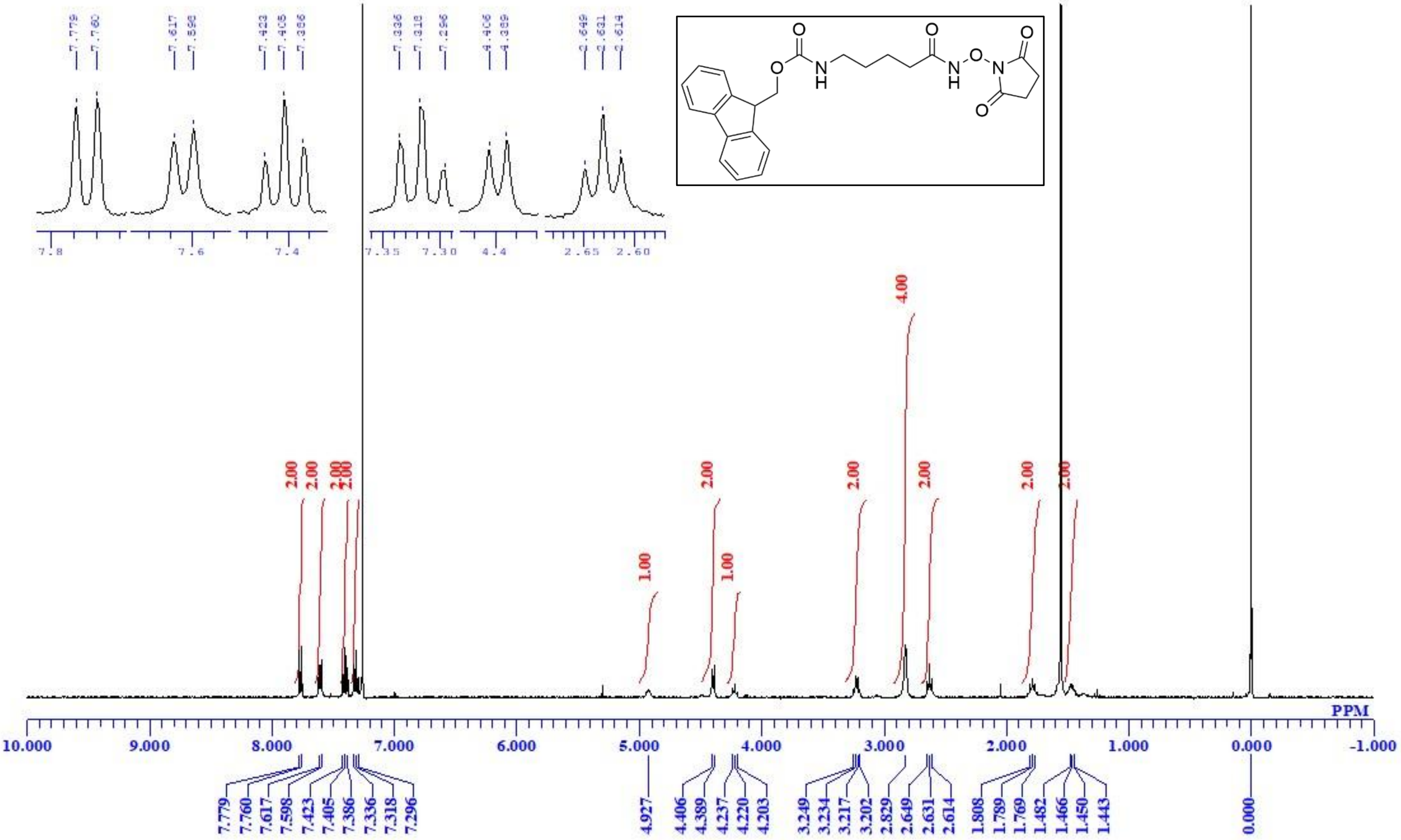

NMR spectrum of **7** (400 MHz, $CDCl_3$)

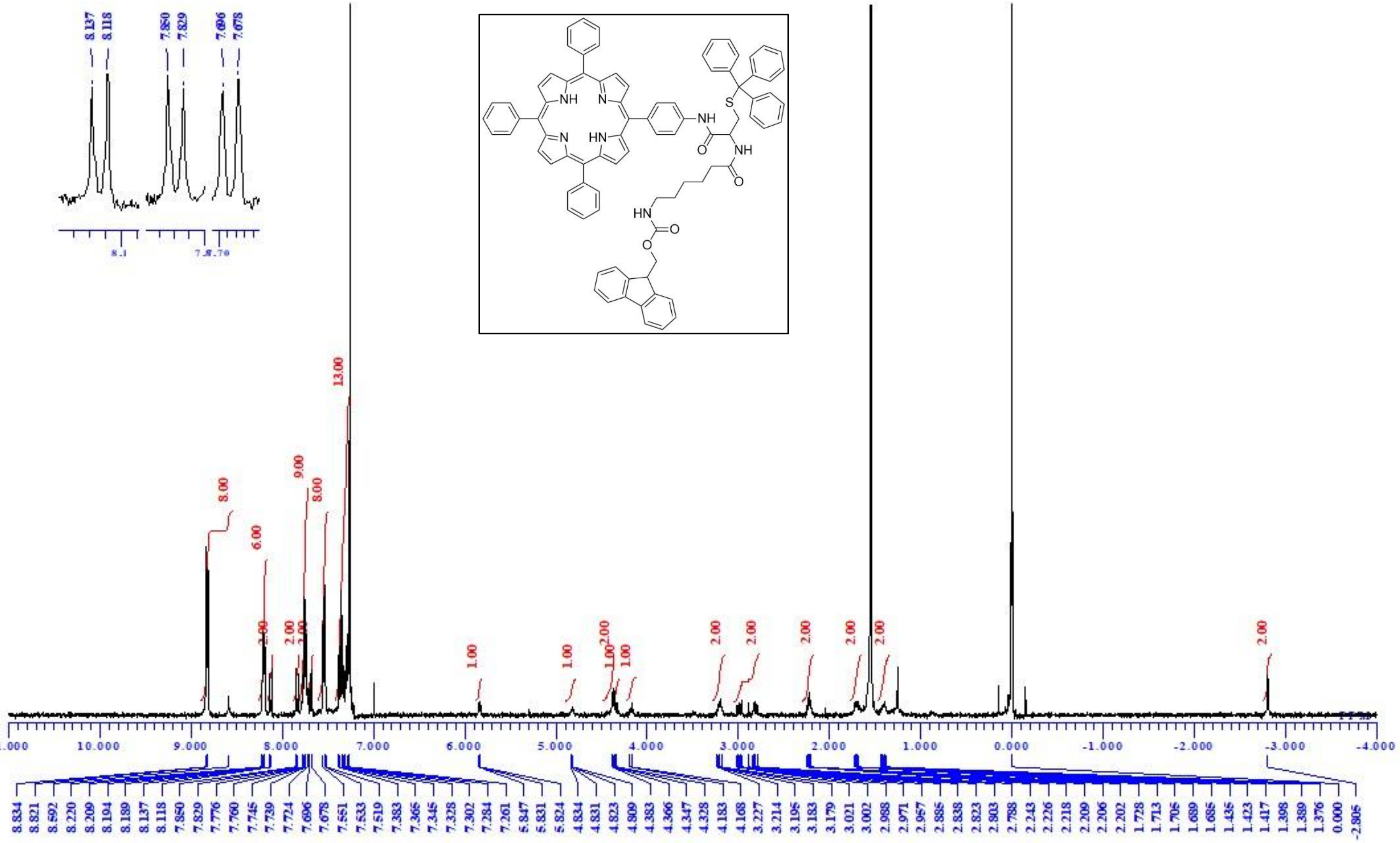

NMR spectrum of **8** (400 MHz, $CDCl_3$)

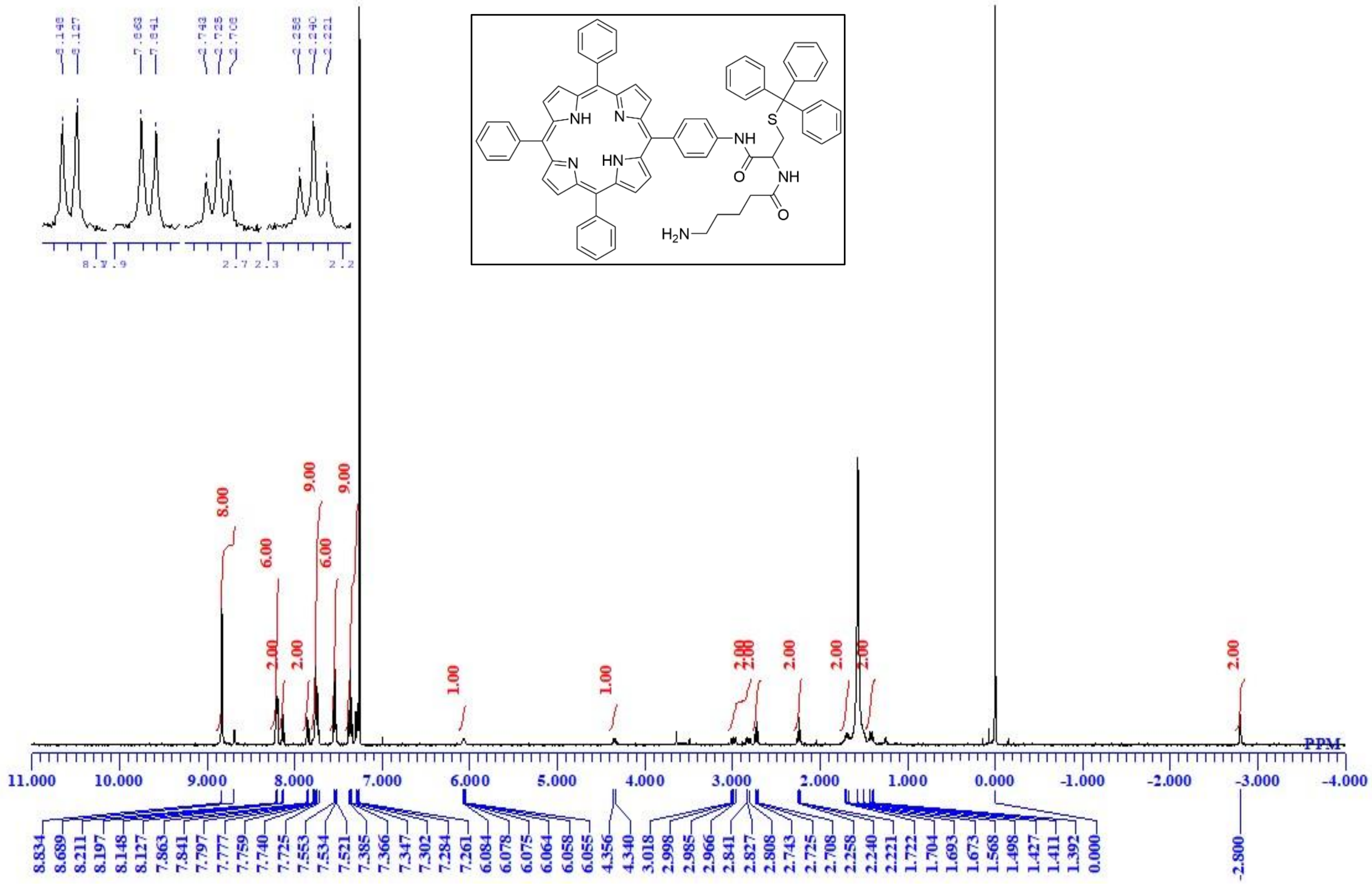

2. MS (ESI+) spectra of 9 - 11.

MS (ESI+) spectrum of **9**

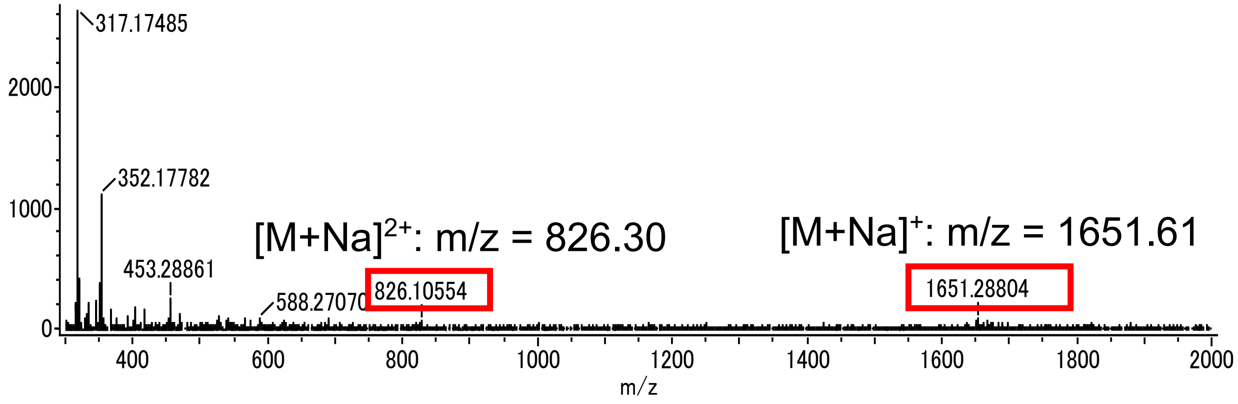

MS (ESI+) spectrum of **10**

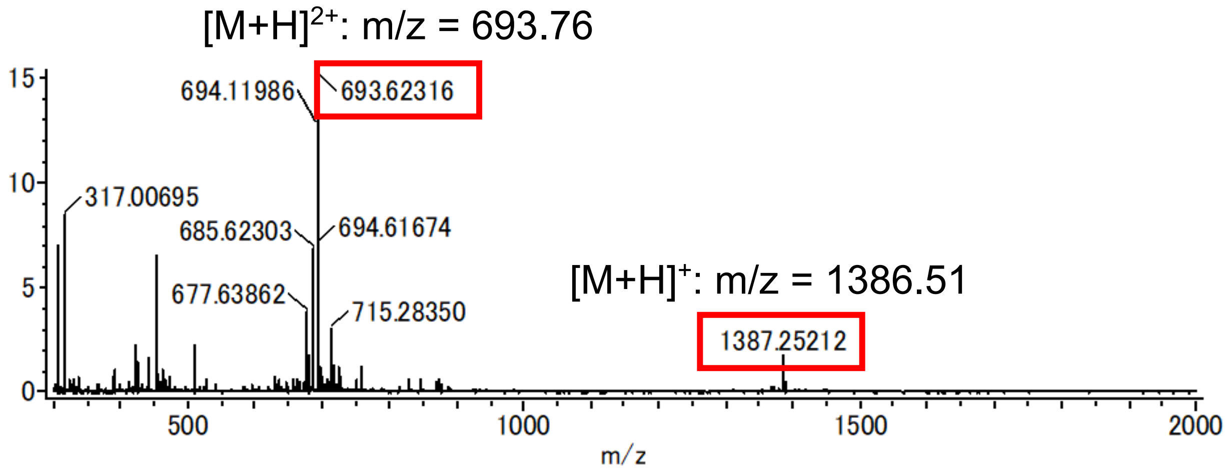

MS (ESI+) spectrum of **11**

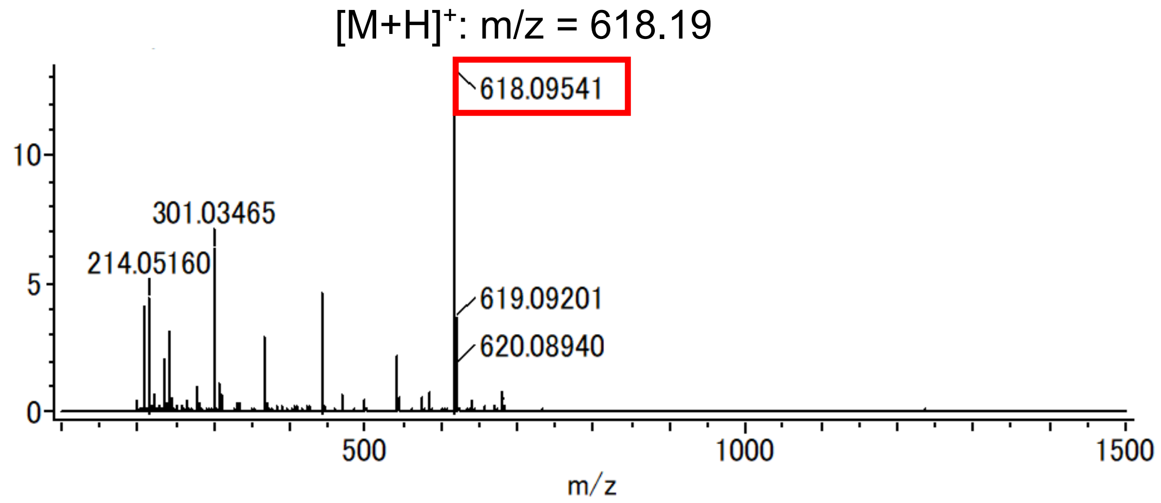

3. HPLC chromatograms of 9 and 11

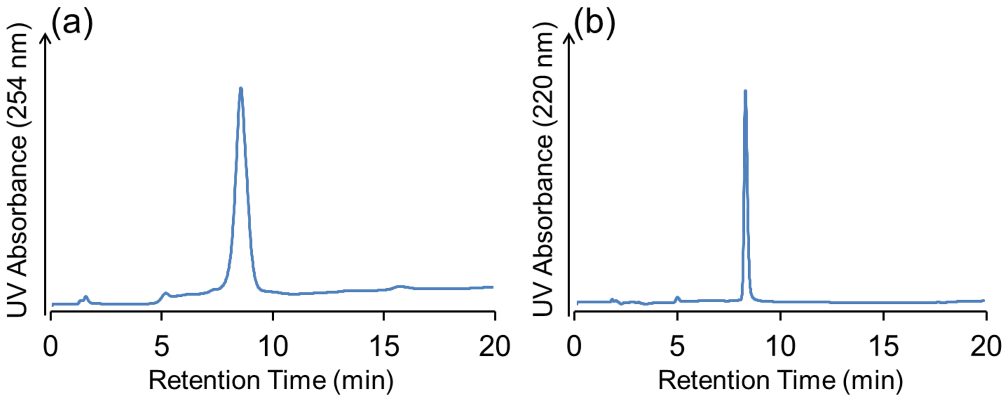

Figure S1. RP-HPLC chromatograms of **9** and **11**. HPLC condition: (a) a flow rate of 1 mL/min with a gradient mobile phase of 40% acetonitrile in water with 0.1% TEA to 60% acetonitrile in water with 0.1% TEA for 20 min. (b) a flow rate of 1 mL/min with a gradient mobile phase of 15% methanol in water with 0.1% TFA to 30% methanol in water with 0.1% TFA for 20 min.

4. Particle size distribution

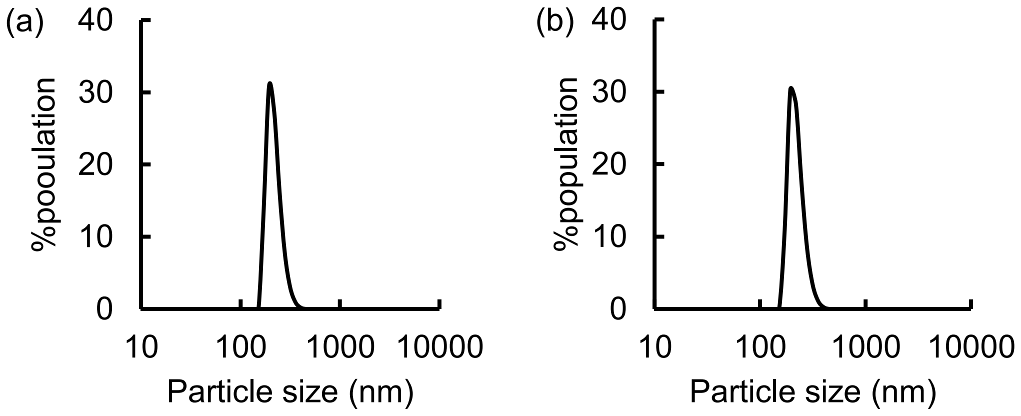

Figure S2. Particle size distribution of (a) DTPA-cysteamine-nanodroplets and (b) DTPA-TPP-nanodroplets. The particle size distribution is presented as a number-based distribution (%population), representing the percentage of particles at each size relative to the total particle number.

5. Cellular uptake

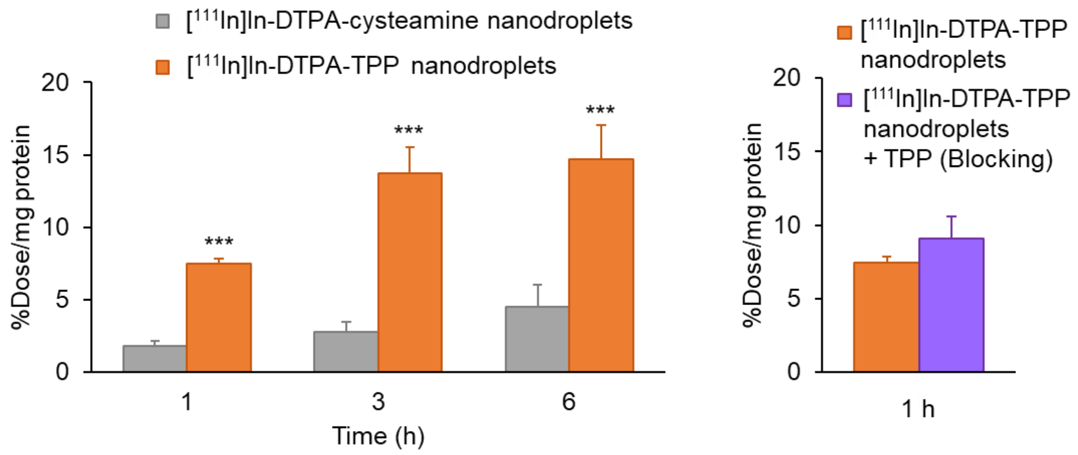

Figure S3. Cellular uptake of [111In]In-DTPA-cysteamine and [111In]In-DTPA-TPP nanodroplets in FBS-containing medium (a) and cellular uptake of [111In]In-DTPA-TPP nanodroplets in the presence or absence of free TPP (b). Data were presented as mean ± SD for four samples. ***$p < 0.001$ vs. [111In]In-DTPA-cysteamine nanodroplets.

6. Biodistribution

Table S1. Biodistribution of [^{111}In]$InCl_3$ at 10 min, 1, and 4 h after intravenous injection in ddY mice

Tissues	Time after injection		
	10 min	1 h	4 h
Blood	13.25 (4.02)	9.84 (2.37)	5.70 (0.83)
Liver	3.28 (0.78)	3.36 (0.72)	4.15 (1.02)
Kidneys	31.67 (6.06)	29.77 (5.10)	34.63 (5.01)
Small Intestine	2.15 (0.34)	2.31 (0.44)	2.26 (0.57)
Large Intestine	1.44 (0.15)	1.47 (0.21)	1.85 (0.36)
Spleen	4.59 (1.31)	4.66 (2.34)	4.12 (0.56)
Pancreas	5.77 (0.64)	5.62 (1.58)	3.15 (0.49)
Lung	11.00 (2.21)	7.69 (2.39)	4.61 (0.77)
Stomach	1.31 (0.47)	1.30 (0.31)	1.22 (0.16)
Heart	5.85 (0.91)	4.61 (1.34)	3.15 (0.45)
Muscle	3.12 (0.89)	3.23 (0.41)	3.08 (0.52)
Brain	0.43 (0.11)	0.41 (0.16)	0.26 (0.06)
Bone	13.64 (1.61)	11.03 (1.98)	13.82 (1.42)

Data are presented as % injected dose/gram tissue (%ID/g). Each value represents the mean ± SD for three to four animals.

7. Detailed SDT data

Table S2. Relative tumor volume (V/V_0) of Colon 26 tumor bearing mice treated with PBS and DTPA-TPP-nanodroplet accompanied by ultrasound irradiation (1 MHz, 2.4 W/cm^2, 50% duty cycle) at 6 h postinjection.

Days post injection	Relative tumor volume (V/V_0)			
	PBS		DTPA-TPP-ND	
	Ultrasound	No Ultrasound	Ultrasound	No Ultrasound
0	1.00 (0.00)	1.00 (0.00)	1.00 (0.00)	1.00 (0.00)
1	1.58 (0.13)	1.45 (0.11)	0.87 (0.77)	1.64 (0.20)
2	2.23 (0.79)	2.04 (0.57)	1.01 (1.32)	2.18 (0.40)
3	2.92 (0.78)	2.71 (0.86)	1.34 (1.75)	3.48 (0.52)*
4	4.42 (1.21)*	3.76 (0.84)	1.59 (2.10)	4.93 (0.95)**
5	5.25 (1.59)*	5.68 (1.70)*	1.73 (2.14)	5.65 (0.95)*
6	7.54 (2.44)*	7.69 (2.55)**	2.29 (2.54)	7.67 (0.77)*
7	11.12 (5.04)**	10.82 (4.19)**	2.54 (2.59)	11.02 (2.05)**

Data were presented as relative values of tumor volume (V/V_0) where V_0 corresponds to the tumor volume on day 0. Each value represents mean (SD) for four mice. Significance was determined using one way analysis of variance (ANOVA) with Tukey's post hoc test (*$p < 0.05$, **$p < 0.01$, ***$p < 0.001$ vs. DTPA-TPP-ND (Ultrasound)).

8. Body Weight (g)

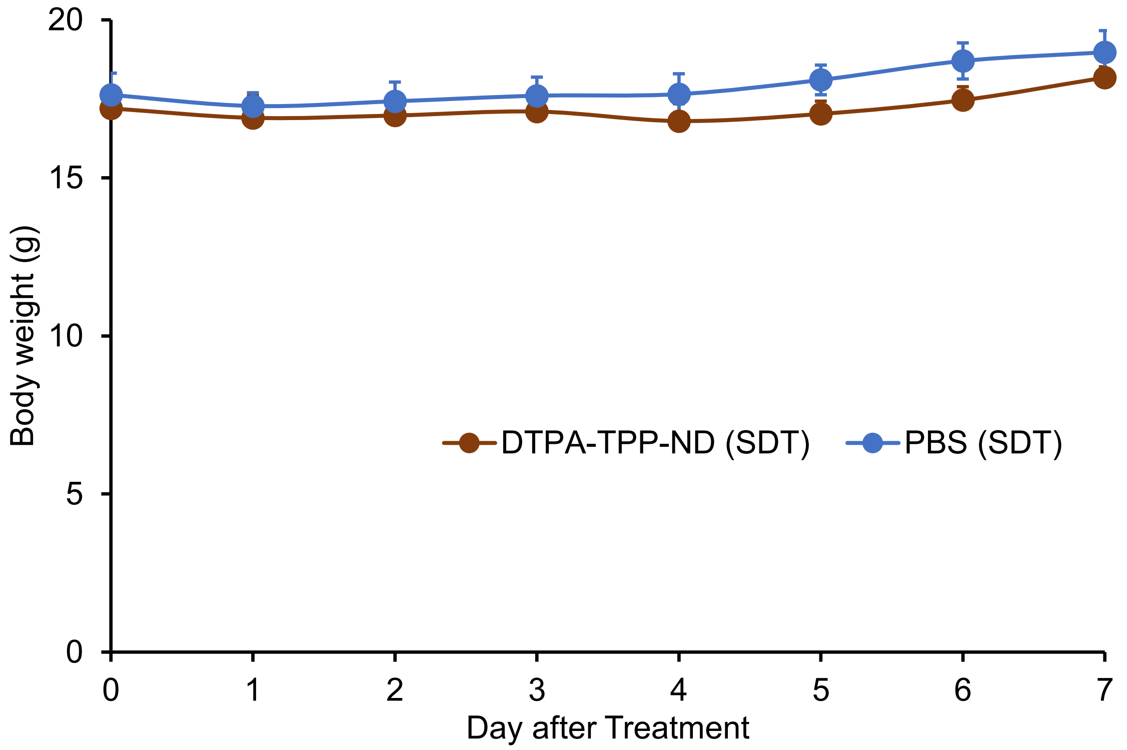

Figure S4. Changes in body weight of mice treated with DTPA-TPP-ND or PBS, both accompanied by SDT at 6 h postinjection. Each value represents mean ± SD for four mice.
